# Spirit of the Foreign Periodicals, &c.

**Published:** 1844-01-01

**Authors:** 


### Spirit of the Foreign Periodicals.

### Last Illness of Dr. Osann: Curious Mistake in Diagnosis.

The name of Dr. *Osann* has been well known, of late years, in German Medical Literature, as being associated with that of his father-in-law, the venerable *Hufeland*, in connexion with one of the most successful journals on the Continent. His case will be found very interesting, and may suggest some useful hints to the practising physician, to warn him against neglecting any means of examination, before he forms his diagnosis.

For many years of his life, he had been subject to frequent attacks of inflammatory catarrh: for these, he was usually bled, with great relief to all the symptoms. The cough was often most harassing and severe, and the sputa were occasionally semi-purulent, conglobate, and streaked with blood. When this was the case, there was generally present a good deal of febrile irritation of the whole system, accompanied with emaciation of the body, tendency to night sweats, &c. As might be expected, serious apprehensions were entertained by many of his friends of the existence of tubercular disease in the lungs. His uncle more than once said, that he regarded him as a *Candidate for Phthisis;* and the patient himself took nearly the same view of his case. The frequent returns of bronchial irritation, the copious expectoration of suspicious-looking sputa, the occasional hæmoptysis, the uneasiness and darting pains in the chest, the hectic irritative fever—these symptoms might well be deemed declaratory of tubercles of the lungs.

But the truth of this diagnosis could not be determined by an exploration of the chest; as the Doctor very strangely refused, on all occasions, to have either percussion or auscultation tried in his case! He said that he felt convinced that neither of these means could be of any real service in directing the proper treatment. "I am always relieved," he said, "by the adoption of antiphlogistic remedies. What good can auscultation do? Supposing that there is some organic alteration in the lungs, the discovery of that should not influence our treatment." So much for a doctor's want of faith, in what he may prescribe to others.

It here deserves to be especially noticed, that none of his medical friends ever entertained a doubt as to the seat of the malady being in the lungs, or for one moment suspected that the heart was at all diseased. And, indeed, how could they?—for there never was any tendency to palpitations, syncope, intermittence of the pulse, dyspnœa on going up stairs or walking quick, &c.

For some time before his decease, Dr. *Osann*'s health appeared to be better established than it had been for several years. He regularly attended to his duties at the University, and seemed to be not at all incommoded even by extra fatigue, although the weather was very cold and trying at the time. On the evening of the 11th of January, he left his home to visit the Dean of the Medical Faculty, Dr. *Jungken;* but, before he could reach the house, he was suddenly taken ill in the street, and carried into the nearest shop.

I found him, says the reporter, supported against the wall, breathing with great difficulty, and harassed with a violent harsh cough. I opened a vein in the arm, but scarcely any blood flowed; and in a minute or two afterwards, life was extinct.

The Dissection was performed by Professor *Froriep*, so that we may fully rely on the perfect accuracy of the following report.

On opening the cavity of the chest, about two or three ounces of a reddish-coloured serosity were found within. At various points, there were old firm adhesions between the costal and pulmonic pleuræ. The parenchymatous tissue of the lungs was crepitant on pressure, sufficiently permeable to air, and of a normal colour, being reddish and of a marbled hue in front, and darker and more purplish behind. In no part were there any traces of extravasated blood, or of hepatization, or tuberculous deposit.

After the most careful examination, nothing, except a minute calcareous concretion—not much bigger than a pin's head—was discoverable in any part of the lungs.

The Cartilages of the Larynx exhibited the appearances of incipient ossification. The mucous membrane of the trachea was more congested than in health; that of the bronchi was quite normal in every respect.

There was no fluid in the pericardium. The heart was evidently considerably enlarged. The hypertrophy was confined to the left ventricle, the walls of which were upwards of an inch in thickness, and very hard and resistant. Its cavity was partly filled with dark-coloured, coagulated blood. The mitral valves were indurated at several points; the aortic valves also were similarly affected.

The real and essential disease, therefore, in this case was (says Dr. *Breyer*, the narrator,) hypertrophy, with dilatation, of the left ventricle. This discovery certainly surprised all the medical attendants not a little, as the very existence of any cardiac lesion had not been once suspected, either by the patient himself, or by any of his professional friends.

It may be here asked, what was the immediate cause of the sudden attack which so rapidly proved fatal?—was it spasm of the heart? or was it cerebral hæmorrhage. We cannot positively determine this question, as the cranium was not examined; but the absence of all apoplectic or paralytic symptoms may be thought sufficient evidence to negative the latter idea.—*Journal de Hufeland.*

*Remarks.*—The preceding is certainly a very curious and instructive case, as shewing—if all the data of the report have been detailed with sufficient accuracy—that the rational symptoms of confirmed pulmonary disease may be present, and yet the lungs may be not at all organically affected. Certainly these symptoms must have been obvious and well-marked in this instance; else an old experienced physician, like *Hufeland*, would not have pronounced his opinion so unhesitatingly; not to mention the unanimous diagnosis of all the other medical attendants.

But the difficulties do not cease here. It is expressly said, that the patient never exhibited any of the usual symptoms of cardiac disorder—palpitations, breathlessness on exertion, irregular pulse, &c. We are however to bear in mind that is it doubtful whether the fatal event at last is to be traced to the state of the heart—for the morbid changes in this organ were far from being considerable, or incompatible with a prolongation of life—or to a cerebral lesion. How far all the symptoms can be fairly attributed to the hypertrophied state of the left ventricle, and the slight thickening of the aortic and auriculo-ventricular valves, it is not easy to determine. Whatever opinion is formed on this and other points of its history, the case is a very interesting one; and not the less so as shewing the insufficiency of the merely rational symptoms, under certain circumstances, to direct the physician to a correct diagnosis. How strange that it remained for a medical man to object to the use of auscultation in his own case; and that in this very case a serious mistake was committed by neglecting the valuable assistance of the stethoscope!—(*Rev.*)

### Death of M. Chervin.

This enthusiastic and indefatigable physician has, after a life of hard toil and poor reward, been recently gathered to his fathers. His countrymen seem to regret his loss more than either he, or any of his immediate friends, could have supposed. As a matter of course, several *eloges* have been addressed to his memory, and all now join in paying respect to his many virtues and talents. "There are some men," says one of his admirers, "whose merits are not discovered, until death has taken them away. Little known, and almost quite overlooked during their lives, an affectionate attention is drawn to their names after their decease; then only, it is found out that a truly good and great man *has* existed in the midst of us. Others again, who may have been surrounded with distinctions and applause while on the busy scene, no sooner disappear than they are entirely forgotten and perhaps are never mentioned again,—like the characters of a play, which are not thought of after we leave the doors of the theatre. If we examine a little into the causes of so marked a difference, we shall most probably find that it is in an especial manner to the amiable qualities of the mind, and to the moral virtues and graces of the character, that our human sympathies are most strongly attached; and that the gifts of intellect, however brilliant they may be, may excite our admiration, but they do not win our love. There is no grace in the character, that more deservedly and generally gains the applause of mankind than unselfish zeal and devotedness of pursuit; but, alas! its value, nay its very existence, is often scarcely known, until the person is taken away from the scene of his labours."

M. *Chervin* was a memorable example of this observation. His whole life was an unceasing struggle, nobly undergone, for an object of universal utility. Fully aware of the difficulties of the pursuit in which he engaged, he yet devoted all the energies of his soul to its unwearied prosecution; he never flagged nor failed in following it out; he spent his time and his property in seeking to arrive at the truth; and at length, as he drew near to the end of his journey, it was with his eye fixed on his favourite theme, that he breathed out his patient spirit. In exchange for such toilsome labours, and for the many dangers, privations, and distresses to which he voluntarily exposed himself, he sought not for that applause of the multitude, which is at once often so hard to win and so difficult to keep. With an entire devotedness of soul to the cause which he had embraced, he seemed to regard himself as the soldier of a single idea, for the triumph of which he was willing to make every sacrifice, alike of private ease and public fortune. He had an enthusiastic passion for the discovery of *truth;* sometimes indeed he carried it to an extravagant length; and it has even been alleged that he would not hesitate to speak *untruths* in its defence! Like most ardent seekers after a single object, he often encountered the shafts of envy, and ridicule. By many he was regarded as a visionary in his dogmas, and as a most importunate *bore* in forcing them upon others. Hence it was that not unfrequently he was advised, even by his own friends, to begin to mind his own private affairs, and not bother himself so much with great objects that only interest governments and nations. But M. *Chervin* was one of those men, whom an instinctive feeling, more sure and powerful than the calculations of reason and vulgar prudence, engages and maintains in an invariable path, but who are often foolishly thought by their cotemporaries to be blind and visionary, because forsooth they look on certain topics with different eyes from the bulk of mankind.

It was to the noble and elevated moral feeling, that ever distinguished M. *Chervin's* career, his disinterested love of whatever is good and true, and his unquenchable and persevering devotedness of purpose, that we may attribute the universal regret that was manifested, by all classes of the profession, at the announcement of his death. He never possessed any fortune, nor rank, nor titles, nor even any high and influential friends; and yet he was respected wherever he went, and commanded the admiration of all our most scientific bodies.

His whole life was devoted to the examination of the Yellow Fever. To discover the laws that regulate its origin and diffusion, to determine whether it be contagious or not, and to point out the sanatory regulations and precautions most necessary for the arrest of this destructive pestilence—such was the great object of all his labours. It was about 1814 that he commenced his laborious and often hazardous enquiries. In the course of that year, he started for New Orleans, with the view of making himself personally acquainted with every thing appertaining to the fevers of that unhealthy locality. He remained upwards of eight years in the United States, and in the course of that time explored not only the entire extent of sea coast from the Gulf of Mexico to the shores of New England, but also the various Colonies and Possessions of the English, French, Dutch, &c. in the West Indies as well as in America. No labour or fatigue ever deterred him from following out his enthusiastic researches, and the minuteness of detail, with which he examined every locality, often excited the surprise and admiration of the resident medical men. With his trunks stored with manuscript descriptions and reports of all kinds, he returned to France, in 1822, to communicate the results of his arduous researches to his fellow-countrymen.

He had scarcely reached his native shores, before he learned that Barcelona and other parts of Spain were suffering from an invasion of what was considered to be genuine yellow fever. The alarm was very general that the pestilence would traverse the Pyrenees and carry its ravages into the heart of France. The Government had already sent a Commission, composed of some of the most eminent physicians of Paris, to examine the disease on the spot, and to suggest what measures should be adopted to check the diffusion of the fever. The result of their enquiries was to favour the idea of its contagiousness; and forthwith various sanatory regulations of a more stringent character than usual were rigidly enforced along the whole line of the Spanish frontiers. M. *Chervin*, whose opinion on this subject was diametrically opposed to that of the Government Commission, thought it a good time to mix himself up with the discussions that were going on everywhere. True however to his invariable practice of examining everything with his own eyes, and unwilling to trust merely to the reports of others, he at once set off for the seat of the disease. Not a spot in Spain escaped his inquisitorial visit. He was engaged in this enquiry for nearly three years. When he returned to France in 1825, he addressed a petition to the Chamber of Deputies, calling upon them to re-examine the question as to the Contagiousness of the Yellow Fever, with the view of relaxing the severity of the Quarantine laws. As a matter of course, the petition was accompanied with a numerous list of documents, illustrative of the important question at issue. The Minister of the Interior called upon the Royal Academy for their opinion upon this grave subject. A committee of nine of its most distinguished members was accordingly appointed to examine all the papers that had been submitted by M. *Chervin*, and to report upon the conclusions which he sought to establish. The enquiry lasted for nearly a twelvemonth. The report was most flattering to M. *Chervin*, and was strictly in accordance with his views—that the existing Quarantine regulations were far more stringent than there was any occasion for, and that the project of building any new lazarettoes was quite unnecessary and most injudicious. This report met with vehement opposition, not only in the Academy itself, but also out of doors.

The Government itself seemed to be rather dissatisfied; for it was generally understood that the project of having more lazarettoes had been already fixed upon. The members too of the Academy, who had formed the official Commission of enquiry in Spain, naturally enough exerted themselves with all their energy against the recognition of views, directly opposed to those which they had published. The Academy certainly did not exhibit a very dignified example of independent feeling on the occasion; for, on finding that their report was not acceptable to the powers that be, they consented to remodel it, and to abate a something of their decided approval of M. *Chervin's* suggestions. He manfully reclaimed against this exercise of official power; but without effect.

While an animated controversy was going on among the *savans* of the French metropolis, the disease (Yellow Fever) was raging at Gibraltar. M. *Chervin*, with his accustomed promptitude, determined to proceed thither without delay. He applied to Government to be sent out in an official capacity, at the same time requesting as a favour that another physician—whose views were entirely opposed to his own—might be appointed to accompany him. The request was acceded to; but, instead of one, two companions were named—M. *Trousseau* by the Government, and M. *Louis* by the Academy. They arrived at Gibraltar in November 1828.

From the period of his return to the time of his decease, M. *Chervin* repeatedly brought the subject of the Quarantine Laws of the country under the examination of the Academy—of which he became a member in 1832—advocating, upon all occasions, the necessity of a gradual relaxation of the existing enactments.

It appears that, towards the close of his life, his pecuniary circumstances were much straitened. He had been long affected with an organic affection of the heart, and had an apoplectic seizure about two years before his decease. He died under the hospitable roof of a friend, on the 14th of August last. The fatal attack seems to have been bronchitis, induced by the cold weather that prevailed at the time. His will is curious; it runs in these words:—

"I have nothing to leave. All that I received from my parents, and whatever I made by a tolerably lucrative practice in the Island of Guadaloupe, has been spent in carrying out those enquiries, in which I have been engaged for the last 27 years, on the origin and mode of propagation of the Yellow Fever, with the hope of inducing the several governments of Europe to remodel their sanatory regulations.

"I have devoted not only everything I possessed to this laborious and expensive undertaking, but also no inconsiderable portion of funds which have been generously supplied to me by the philanthropic assistance of friends. My labours in this field of enquiry having been the means of diffusing important information, which has benefited my country not a little, I venture at this solemn moment to express a hope that France will re-imburse those of her patriotic citizens, who have enabled me by their liberality to carry out the enterprise, in which I was so long engaged.

"I owe certain sums to my landlord for the rent of my house, and to the printers of my writings for work done. I trust that the Minister of Trade may be pleased to purchase the impression of several manuscript papers on the subject of the Sanatory Laws and Quarantine Establishments of the country.

"My desire is that my honourable friends MM. *Reveillé-Parise* and *Londe*, Members of the Royal Academy of Medicine, may be pleased to charge themselves with superintending the publication of these writings. To them I leave all my books and papers."

We trust that this touching appeal to his country will be responded to as it deserves.

---

### M. Reveillé-Parise on Health and Disease.

The perpetual Secretary of the Academy of Medicine is a learned and very amiable man. Most of his writings have a philosophic and moral cast of thought, and will therefore be more valued by the lover of contemplative study, than by him who is engaged in the practical duties of a fatiguing profession. It is well, however, occasionally to have our attention diverted from the ever-recurring minutiæ of special disease, and let it muse for a time on the generalisations of a more comprehensive pathology.

"Although the causes of diseased action appear, on first consideration, to be almost innumerable, it is nevertheless quite possible to reduce them all to three principal categories—Wounds, Poisons, and excess of Excitement, moral or physical. Now these causes may act either separately or simultaneously; the latter case is the most frequent, as well as the most dangerous. The reason of this is obvious. Man avoids as much as possible the reach of the first two; but often, too often, he voluntarily exposes himself to the hurtful influence of the third—forgetting, as he blindly does, that, although he may succeed in augmenting the intensity of pleasurable excitement by the use of undue stimulants, he cannot increase the organic capacities of his constitution to bear it without injury. The reckless search after pleasure is the rock, on which health is most frequently shipwrecked. It is the perpetual *besoin* of exciting sensations, which perhaps have already become blunted from previous inordinate stimulation, that is the root of very much of the worst suffering to which civilised life is subject. This is the secret cause of the corrosive influence of sibaritic indulgence and sensual pleasures. The body is more excited than Nature intended it should be; and, by a law which she herself has impressed upon the system of man, the excessive gratification of any appetite induces an increased desire for satisfying its demands, while, at the very same time, the powers of the instrument are proportionally diminished. And yet no experience will convince mankind of the truth of all this. Every day we may see proofs of the danger of inordinate organic excitement; but, alas! these proofs are heedlessly passed by all of us, as if the danger could never reach ourselves. Let us for a moment simply ask, what is the cause of such infatuation? It is two-fold—on the one hand, there is the desire for continual and ever-renewed excitement; and, on the other, the circumstance of the dangerous consequences of such over-excitement being not immediately felt. The penalty is not paid at the moment; and weak man shuts his eyes to the future—"Because sentence against an evil work is not executed speedily, the heart of man is set in him to do iniquity." *Montaigne* has wittily remarked, that the reason, why a sane man will not put his hand into the fire, is only because he knows that he should *at once* suffer for his folly. Alas! this is not the case with the equally real and dangerous experiment of indulging unbridled passions; although the penalty, it is well known by us all, must be suffered at some time or another.

How truly has *Plutarch* said, that "We, in our ignorance, call, by the name of *delay*, the time that divine justice is but lifting its hand to give the heavier blow." This beautiful reflection is strictly applicable to the laws which regulate the organism of man. There is indeed a *Nemesis* which, like the fabled one of antiquity, may delay its vengeance for a time, but which will assuredly not allow the guilty to escape altogether. And how can it be otherwise? The punishment is the result of the very laws, which rule the organism; health is the blessing of obedience to them, and suffering and disease are the necessary consequences of their violation.

How often do we wilfully forget that there is a monitor within, appointed by Nature herself, to give us warning of the coming danger, when the *physiological law*, which we are now endeavouring to expound, is infringed or disobeyed—we allude to the sensation of Satiety! The very power, which the economy enjoys to habituate itself to different impressions, is like a remedy that is held in reserve by Nature against most of those accidental evils, which would otherwise be induced by various external agents. In one case it may act as a mitigator of pain, the perception of which becomes less acute, whenever it has lasted long; while, in another case, it serves as a counterpoise to the ever-recurring desire for renewed excitement. However this may be, we certainly find that, when excitement reaches to a certain degree, or is repeated a certain number of times, the sensation induced becomes gradually more and more blunted. The piquancy of novelty, and the freshness of the impression, become less and less, until they finally cease; and then, if the stimulation be continued, a positive disrelish or even disgust takes the place of what was once a pleasurable sensation. What a benevolent arrangement is this of Nature to prompt us to abandon, or at least to moderate, what otherwise must eventually injure! And yet how often is it wilfully neglected; and foolish man resorts to merely increasing the dose of the stimulus, in the vain hope of renewing his jaded appetites. Thus it is that the tone and natural vigour of certain organs are gradually wasted, until perhaps they cease to do their duty at all, and death is the result of the suspension. So many errors are daily committed under the impression that, because no injury is directly or immediately sustained by the individual who disobeys the laws of nature, he escapes altogether. The penalty, although tardy, is sure to be demanded, and the body will ere long evince the fruits of the disobedience.

We may therefore assume it as a most incontrovertible fact that the intensity and continuance of impressions, even with the tolerance acquired by habit, cannot be prolonged beyond a certain degree. They are sooner or later arrested by the penalty of suffering. But there are men, and these not a few, whom even this penalty will not deter from their infatuated course; they cannot endure the feeling of *ennui*, and they must have excitement at any cost. How true is this of the smoker, the drunkard, and the debauchee. The same remark holds true of the passions of the mind. There is a strict alliance and sympathy between the disorders of the flesh and of the spirit.

*Rochefoucauld*, with his characteristic severity and point, has said that, 'although there are many women who have never had an intrigue, there is not one who has had only one;' and a lively writer of the present day very truly remarks that, 'the punishment of those, who have loved women to excess, is, that they never cease to love them.' Such is the force and tyranny of inordinate and irregular indulgencies.

Another, not less striking, consequence of the irrepressible desire, or *besoin*, of the system for renewed stimulation, when any organ has been excited beyond a just degree, is that the degree of the physical impression is ever dependent upon the state of the organism. Not only is there something or another always wanting—for he that has ten *besoins* is not happy, if nine only be gratified—but the degree of enjoyment, actually obtained, is always below what was anticipated and hoped for.

Often has the philosophic physician an opportunity of observing the unhappy struggle between the dreams of fancy and the realities of life! Man is so constituted that he should never pitch his expectations of pleasure very highly; for assuredly he will be disappointed when the very object or attainment, for which he may long have panted with most feverish excitement, is given him to enjoy. The immediate organic effect will be deceptive, and at best it must be transitory; the pleasure *felt* will never equal the pleasure that has been *imagined*, more especially when the first edge of our sensations has once been blunted. *Cæsar* said of the empire of the world, 'Is this all?'; and we read that *Vespasian* expressed utter weariness at the length of his triumph. It is not in worldly hopes and earthly pleasures that the mind of man can ever obtain its full draught of satisfying enjoyment; it must soar above the things of time, and hold communion with objects, lofty and immortal as itself. Let not the reader suppose that this train of thought is quite foreign to the pursuits of medical life: far from it. Unless the physician has learned to mark and watch the workings of the soul, and their intimate and often immediate connexion with the curious machinery of the body, he has studied but one-half of his profession; and he will therefore often fail to give that relief to suffering humanity, which should ever be the great object of his thoughts to administer.

It was a saying among the ancients that *acute* diseases were sent from Heaven, and that *chronic* ones came from ourselves. There is a good deal of truth in this Sacro-medical axiom. The arrow is winged from above; but it is our own hand which poisons it. We thus see that in all our enquiries we must bear in mind the nature and constitution of man, the vital acts of his body, and the laws which regulate and direct them. When we remember how very few of those persons, who have once given themselves up to violent and inordinate excitement, stop at the point which prudence dictates, we cannot be surprised at the amount of bodily and mental suffering that exists among all classes of society. Frail man! urged on by the instinctive law or attribute of his system, that every organ which is over-excited becomes thereby more excitable, he allows himself to be led along into excesses, the hurtful effects of which are, although slow and perhaps unperceived, infallible and unerring. Over-excitement—*mater sæva cupidinum*—is the prolific source of the worst diseases, to which his body is liable. To say metaphysically that 'the flesh is weak,' is to express at the same time the *besoin* of the organism for excitement, and the danger of this being carried too far; and, if the flesh is weak, 'the spirit is not always ready;'—in other words, the instinctive impulses are often too much for the force of the will and intellect to withstand.

The preceding observations are as applicable to excessive study and to over-violent or too-protracted bodily exertion, as to inordinate stimulation of the merely organic functions. The ascetic devotee of science or literary pursuits may transgress the laws imposed by Nature, quite as much as the reckless drunkard or the profligate debauchée. There is a passion for knowledge, that, when carried to excess, may damage the health as deeply as indulgence in more ignoble pursuits. The physiological law is the same in both cases; and hence the violation of this law is followed by similar consequences. The brain may be over-excited by intense or over-prolonged study, just as the stomach is apt to be by excessive or renewed potations.

But let us stop here for a moment and reflect how beneficient are the arrangements of Nature. Although the over-working of the mind may be followed by exhaustion for a time, it never loses its taste for the delights of scientific and literary pursuit; the very indulgence, if not carried too far, begets a keener relish for more; and thus, till the close of life, it may be ever renewing the delights and recreations of its youth and early associations. How different from the encreasing *ennui* and distaste to the jaded appetite of the sensualist! Each year takes off a something from the *verve* of his former desires; every pleasure is stale; the perceptions are blunted; and the very excitement, such as it is, is inevitably followed by increased exhaustion, and perhaps by actual suffering.

M. *Parisé*, after commenting very eloquently on the ill effects of mental anxiety and disquietude on the state of the bodily health, very justly remarks that "certain diseases, as aneurisms of the heart, cerebral congestion, various morbid affections of the nervous system, insanity, &c., are infinitely more frequent now, than they were in former ages, especially in large cities. All this is more from mental than even from corporeal dissipation. Unless the passions are more than usually strong, and the judgment is very weak at the same time, most men find that, as they advance in years, they must put a check upon indulgence in such sensual excesses, as manifestly injure their health. But this is not the case with the o'er-mastering feelings of ambition and avarice. As years increase, these increase; and the very food, on which they feed, but whets their appetite for more. Age cannot check it; disease and infirmities seem to make no change; and it is Death only that can say, 'Hitherto thou shalt go, but no further.'

* * * When *Montaigne* says, 'Defiez-vous de la trahison de vos plaisirs,' he but expresses, in another form, an important physiological maxim, and illustrates the truth of what I have already remarked, that medical science only renders sure and positive what philosophy suggests as probable. Both alike warn us of the dangers of transgressing those rules, which Nature has appointed; shewing us, on the one hand, how our bodily and mental comfort may be secured by obedience, and on the other, how inevitably we are made to suffer by acting in opposition, to her arrangements."—*Gazette Medicale.*

---

### Orthopædic Controversy in Paris.

A very angry dispute has, for some time past, been going on between M. *Guerin* —*the* orthopædist (par excellence) of the French metropolis—and M. *Malgaigne* and some other members of the French Academy, relative to certain statistical reports of alleged cures of various deformities, communicated by the former gentlemen to the Bureau des Hôpitaux. Many of his *confreres* had long suspected that the doctor of the establishment of La Muette, (near Passy, we believe,) in his overflowing admiration of subcutaneous Myotomy and Tenotomy, must surely have somewhat exaggerated the marvellous success which attended all his operations. Wry-necks, crooked backs, bent knees, club-feet, stiffened fingers and toes—not to mention squinting and various other kinds of deformity—seemed to be got rid of in a twinkling, and scarcely ever did we hear of a case that was not cured. Our readers are aware that M. *Guerin* is not only very adroit, but also most persevering and courageous, in carrying out his brilliant 'decouverte' (invention, we should rather call it,) of dividing muscles and tendons, without making more than a puncture through the integuments. On one occasion he divided no fewer than 40 muscles or tendons in a single case, and the result was, as a matter of course, that the patient was 'parfaitement gueri.' The success of his operations was so unvarying, that many of the medical men of Paris began to have their doubts as to the complete veracity of the reports; and M. *Malgaigne* in particular seems to have set about a strict enquiry, with the view of ascertaining the exact state of the question. His statements are certainly far from being in accordance with the assertions of M. *Guerin.* Many of the cases reported as cured have been, it is said, scarcely, if at all, benefited by the treatment adopted at La Muette; and, in several instances, the same patient figures more than once on the successful list. We need scarcely allude to another charge that has been brought by some against M. *Guerin.* It is alleged that many of the gratuitous patients, sent by the Bureau des Hôpitaux to the Doctor's Establishment, have been made to pay for the plaster-moulds that have been taken of their deformities and the apparatus which have been supplied, and that M. *Guerin* has been no loser on these occasions. As we have no opportunities of knowing the truth of this charge, it is better to keep it entirely out of our view, and to confine ourselves solely to the medical part of the accusation. We were not, we must confess, at all surprised when we first read of the charge of bad faith being brought against the orthopædic doctor.

There has always been such extravagance in his published reports, such unheard-of suscess in everything that he set his hand to, such marvellous cures effected by a *coup-de-main,* and withal such silly affectation of scientific profundity, that we long ago settled in our own minds that all that he said was not gospel. That his reports of successful cases have unquestionably been *inaccurate,* cannot, we suppose, be now denied. He may indeed have imposed upon his own credulity, and it may be that thus he has been unconsciously led to mislead the minds of others by his statements. But then there has been all throughout so loud and magniloquent a boasting, that we almost hesitate to give him the benefit of this charitable interpretation.

While unwilling to exculpate M. *Guerin* from, at least, the charge of most unworthy indiscretion, we must lay part of the blame to the charge of the Royal Academy itself, as some of the practices, connected with the proceedings of this learned body, have always appeared to us to open the door for much accidental, if not wilful, misstatements. We allude more particularly to the common occurrence of members reporting cases, while still under treatment. Nay, not only this; but, every now and then, we read of successful operations being pompously announced on the very evening perhaps of the day, on which they were performed. Sometimes indeed the case is rendered still more dramatic, by the patient being exhibited to the Academy before and after the surgical treatment—all seemingly for the purpose of giving the operator, and the other members, an opportunity of saying something about themselves.

M. *Guerin*, as might be imagined, is especially fond of such exhibitions. For example, we find, in the account of the proceedings of the Academy on the 18th of July last, that he exhibited a patient, affected with lateral deviation of the spine, on whom he had a few days before performed the operation of tenotomy. "I have," said he, "in this case, divided the *longus dorsi* and part of the *sacro-lumbaris* on the right side, and the spinal portion of the *longus dorsi* on the left. As the patient, at the present moment, wants part of the muscles that are necessary for the act of standing, he requires to be supported." "The section of these muscles," the learned doctor continued, "not proving quite sufficient in itself for rectifying the deformity, it is necessary in this case to have recourse to an apparatus, for the purpose of keeping the parts in a normal attitude. Here, therefore, we must act, as we do in cases of club-foot, where we first divide the tendons that are at fault, and afterwards retain the limb motionless and in a proper position for some time. I repeat that this patient is not yet cured, and that I have exhibited him only to prove how innocuous the operation of tenotomy is, and to shew its immediate effects." So much for the *show-man;* now for the remarks of some of his auditors. M. *Bouvier* (a rival orthopædic doctor, we believe) gets up and tells the learned Academy that all, that M. *Guerin* has been saying, is mere fudge; that the patient is not a whit benefited by having had his dorsal muscles cut; and that all the *seeming* improvement in the straitness of the back is altogether owing to the person being shewn off in the reclining position.

As a matter of course, human endurance cannot bear such scandal. Up starts the operator, and denounces, as so many falsehoods, the observations of his learned *confrere.* At the same time, he gives vent to certain pathological axioms on his favourite subject, which we cannot do better than give in his own words. 1. "I regard," says he, "that lateral deviation of the spine is a sub-luxation of the vertebræ, and therefore remediable only in one way, by effecting a certain reduction of the displacement in question;—2, that this reduction is indispensable to neutralise, in part, the influence of the vertical action of the weight of the head and upper part of the body;—3, that, in this respect, deviation of the spine is exactly in the same condition as club-foot, which does not become rectified of itself after the division of the contracted tendons, but which requires to be kept mechanically extended afterwards for some time."

The analogy, which M. *Guerin* here seeks to establish between lateral deviation of the spine and club-foot, is, in our opinion, quite faulty and fallacious. There is no permanent contraction of any of the muscles in the former disease, as there is unquestionably in the latter. Is not the mere fact of the curvature *redressing* itself when the person is in the horizontal position, and therefore when all the weight of the head and neck is withdrawn from the weak spinal column, a sufficient evidence of this fact? We think it is.

Club-foot is never the result, as far as we know, of weakness in the affected part. But it is unnecessary to say more on this subject at present, as the pathological reasonings of M. *Guerin* are much less likely to be adopted than his manipulations. His practice of dividing some of the spinal muscles has been tried by one or two surgeons in this country; and, it may be, with a certain amount of success in a few cases. But we must protest against the operation being at all applicable, in a general point of view, to the treatment of lateral curvature of the spine; for the practice is based on erroneous principles, and cannot therefore be successful in the majority of instances.

What has been said above will serve, among other examples, to guard the medical reader from hastily yielding his belief to all the bold assertions of popularity-hunting doctors, whether in our own country or on the continent. There is no end to clap-trap novelties in our profession. On the one hand, we have men who cure all deformities, whether of the eyes, or the limbs, or the back, by cutting muscles and tendons, and would make us believe that Stammering and Deafness may be got rid of almost *instanter* by snipping off a bit of the uvula or tonsils—(by the bye, what is Mr. *Yearsley* doing now-a-days?)—and on the other, we have at the present moment quite a bevy of *claqueurs*—including metropolitan surgeons, and Irish-dubbed knights—discoursing most learnedly on the merits of Hydropathy, and hospital physicians and country parsons proclaiming the wonders of Animal Magnetism.

Truly this is the age of rampant quackery; and high time it is that those, who value the dignity of professional credit, should unhesitatingly denounce the folly or the knavery—for assuredly it is one or the other—of all such practices.

### Illustrations of Diseases from the Latin Classics.

It has often occurred to us, in our musings on Medical Journalism, that a series of interesting, and at the same time not uninstructive, papers might be written in the way of Commentaries on numerous descriptive passages from the writings of the most distinguished classics of all ages, to show how much light and illustration may be thus thrown, with pleasing effect, on various topics of professional enquiry. No one can have read the works of our immortal dramatist with any degree of attention, without having noted the extraordinary truthfulness of many of his allusions to the workings alike of our bodies and minds, under the influence of disease as well as in the energy of health. Sir *H. Halford,* it is well known, selected a passing remark of Hamlet as the theme for a very ingenious professional Essay; and, indeed, there is scarcely a play of Shakspeare that will not afford more than one such example. But it is not from English writers that we are going to select our quotations; the master poets of Rome are to furnish our examples on the present occasion. It is but fair to say that, for most of them, the reader is indebted to the literary taste of a writer in the Gazette Medicale of Paris. They form the staple contents of an ingenious *feuilleton* article in that well-established periodical. Our chief motive, for introducing them to the notice of our own countrymen, is to exhibit one of the traits of continental journalism, and to shew how agreeably some of our French brethren know how to blend literary amusement with professional information. They may fairly challenge our applause, by quoting to us the Horation precept,

> Omne tulit punctum, qui miscuit utile dulci,
> Lectorem delectando, pariterque monendo.

The description of the Plague by *Lucretius* is considered, by the best judges, to be the finest part of his philosophic poem *De Naturâ rerum.* It is characterised by a strict adherence to truth, and a most faithful narration of all the striking features of this terrible disease. There is no fanciful embellishment, nor exaggerated colouring introduced into the picture. This therefore owes most of its impressiveness to the very simplicity, with which the artist has managed all the details. In this respect it affords a striking contrast to the more ornate, and consequently the more feeble, portraiture of the plague of Latium given by *Ovid,* in the 14th book of the Metamorphoses.* Let us select one or two passages from the old poet's truthful description. How forcibly does he paint the symptoms of the disease, with his characteristic severity and point, in the following lines!

Principio, caput incensum fervore gerebant,
Et duplices oculos suffusâ luce rubentes:
Sudabant etiam fauces intrinsecus utro
Sanguine, et ulceribus vocis via septa coibat;
Atque, animi interpres, manabat lingua cruore,
Debilitata malis, motu gravis, aspera tactu.
Inde, ubi per fauces pectus complerat, et ipsum
Morbida vis in cor mæstum confluxerat ægris,
Omnia tum verò vitaï claustra lababant.
Spiritus ore foras tetrum volvebat odorem,
Rancida quo perolent projecta Cadavera ritu;
Atque animi prorsum vires totius, et omne
Languebat corpus, leti jam limine in ipso;
Intolerabilibusque malis erat anxius angor
Assiduè comes, et gemitu commixta querela;
Singultusque frequens noctem persæpe, diemque,
Corripere assidue nervos, et membra coactans
Dissolvebat eos, defessos ante, fatigans. *Lib.* vi.

The burning headache, the red suffused eye, the throat ulcerated and oozing out blood, the tongue rough and parched, the corpse-like fætor of the breath, the prostration of all the vital energies, the sense of anxiety and anguish about the precordia, the constant moaning and hiccup—all these symptoms are powerfully and faithfully delineated. To do the poet justice, it would be necessary to make large and numerous quotations from his graphic description of the pestilence. He describes it as coming from Egypt—that hot-bed of epidemic disease in all ages—and does not forget to tell us, that its invasion is always preceded by an alteration in the state of the atmosphere, by the exhalation of effluvia from the earth, and by the simultaneous operation of certain sidereal influences. He mentions, also, the eruption of gangrenous tumors or buboes in different parts of the body, and the extensive disorganisation of the tissues, where these make their appearance. In the following passage he enumerates with great accuracy, and an almost professional skill, a variety of details, which cannot fail to interest every medical reader.

Multaque præterea mortis tum signa dabantur:
Perturbata animi mens in mærore, metuque;
Triste supercilium, furiosus vultus, et acer;
Sollicitæ porro, plenæque sonoribus, aures;
Creber spiritus, aut ingens, raroque coortus;
Sudorisque madens per collum splendidus humos;
Tenuia sputa, minuta, croci contincta colore,
Salsaque, per fauces raucas vix edita tussi.

The style and diction of *Lucretius* acquire a truly remarkable precision, when

---

* *Lucretius* however has, it should be stated, borrowed very largely from the wonderfully graphic account of the plague at Athens, left on record by the historian *Thucydides*.

he comes to paint the close of the frightful scene. One might almost suppose that the following lines were a translation from a passage in the writings of Hippocrates:

——————— ad supremum denique tempus,
Compressæ nares, nasi primoris acumen
Tenue; cavati oculi; cava tempora; frigida pellis,
Duraque; in ore jacens rictum; frons tenta minebat:
Nec nimio rigida post strati morte jacebant,
Octavoque fere candenti lumine solis,
Aut etiam nonâ reddebant lampade vitam.

*Ovid*, in his well-known description of a pestilence in the 7th Book of his Metamorphoses, tells us that, for several months before its eruption, the weather had been hot, gloomy, and oppressive, the wind being the lethiferous South. At first, the disease was strictly *epizootic, i. e.* affecting the lower animals, even to the savage denizens of the forests. The air is described as becoming vitiated with the effluvia from the putrifying dead bodies, which

——————— dilapsa liquescunt,
Afflatuque nocent, et agunt contagia latè.

At length the pestilence attacks mankind, alike in the country and in towns. We quote the description of the leading symptoms: though vigorous, it has not the minute and truth-like touches of the elder poet.

Viscera torrentur primo; flammæque latentis
Indicium rubor est, et ductus anhelitus ægrè.
Aspera lingua tumet, tepidisque urentia ventis
Ora patent; auræque graves captantur hiatu.
Non stratum, non ulla pati velamina possunt;
Dura sed in terrâ ponunt præcordia; nec fit
Corpus humo gelidum, sed humus de corpore fervet.

His description would apply better to a case of acrid poisoning, than to one of pestilential fever. Much of what remains in the original is far too fanciful to be at all like Nature. No doctor, in the present day at least, ever heard of fever patients leaving their beds, and rushing to wells, rivers and fountains to quench their intolerable thirst. Some of *Ovid's* drank so much that

——————— graves nequeunt consurgere, et ipsis
Immoriuntur aquis: aliquis tamen haurit et ipsas.

Let us now compare the above descriptions of the disease in the human being with that which the prince of Latin song has given us of the Autumn pestilence among cattle. Hear what he says of the horse, when the invisible foe has lodged the poison in his frame.

Labitur infelix studiorum, atque immemor herbæ
Victor equus, fontesque avertitur, et pede terram
Crebra ferit; demissæ aures; incertus ibidem
Sudor, et ille quidem morituris frigidus; aret
Pellis, et ad tactum tractanti dura resistit.
Hæc ante exitium primis dant signa diebus.
Sin in processu cæpit crudiscere morbus,

Tum vero ardentes oculi, atque attractus ab alto
Spiritus interdum gemitu gravis; imaque longo
Ilia singultu tendunt; it naribus ater
Sanguis, et obsessas fauces premit aspera lingua.*

The last four lines are admirable, and present to the eye quite a picture of the dying animal. The reader will not fail to notice also the description of the state of the skin in the third and fourth lines: it might be applied, with great truth, to many cases of bad fever in the human being. Towards the close of the narrative, the poet alludes to the dangerous consequences of handling the bodies of the animals that have died of the disease:

Nec tondere quidem morbo, illuvieque, peresa
Vellera, nec telas possunt attingere putres.
Verum etiam invisos si quis tentaret amictus,
*Ardentes papulæ*, atque immundus olentia sudor
Membra sequebatur; nec longo deinde moranti
Tempore, contactos artus sacer ignis edebat.

With one short quotation from Juvenal, on the spreading of disease by contagion, we close our illustrations for the present.

—————————— Contagio labem
Et dabit in plures: sicut grex totus in agris
Unius scabie cadit: ——————
Uvaque conspectâ livorem ducit ab uvâ.

---

LETTER FROM M. BISCHOFF TO M. BRESCHET ON FECUNDATION.

"In two memoirs which I have recently published—A History of the Development of Mammiferous Animals, and a History of the Ovum in the Rabbit—I have endeavoured to determine with greater accuracy, than had been done previously, the exact period when the ova pass from the ovary into the oviduct, more especially in the dog and rabbit tribes.

In the first place I observe that, according to the results of most accurate and generally-received experiments, there is no *necessary* mutual connexion, in any tribe of animals, between the escape of the ova from the ovary and the acts of coition and fecundation. Everywhere we find that the ova are developed and matured in the female animal, and may become detached from the ovary and separated from its body, quite independently of any participation of the male animal. In a great number of cases, we know that the fecundation of the ova by the male does not take place until after the expulsion of the ova, not only from the ovary, but even from the body of the female; while, in numerous other examples, we observe that, although the act of fecundation takes place within the body of the female, the development, maturation, and detachment of the ovum in these animals are often effected without any copulation: the ova however being then not susceptible of ulterior development. The acts of coition and fecundation are, so to speak, only accessory and accidental, as far as the formation,

* Let our readers compare this poetic narrative with the description of some of the diseases in the horse, given in the article on Veterinary Medicine in the present Number. It will then be seen how true to nature Virgil has kept.

maturation, and expulsion of the ovum are concerned; but they are essentially necessary for its complete development. Such is the case in the human species as well as in the lower animals—viz: that ova may be detached, at periods of greater or lesser regularity, from the ovaries, quite independently of any communication with the male.

I am convinced, by the results of very numerous experiments on bitches and rabbits, that, after the ligature and extirpation of the uterus, the phenomena of generation invariably take place thus far, provided the ovaries and Fallopian tubes have remained uninjured. The animal will rut at the usual period, and copulate with its male, and the ova will be developed in and separated from the ovaries, reaching the oviduct as in a state of health; but, not being fecundated, they never, as a matter of course, can exhibit the phenomena of embryonic evolution.

I have also satisfied myself that, just as the ova may be formed and matured in the female independently of any co-operation on the part of the male animal, so the seminal fluid of the latter pursues its usual course independently of the ova in the female. In two bitches, which were carefully examined a few days after copulation, I found several detached and fecundated ova in one of the Fallopian tubes, while in the ovary on the other side there was not any tumefaction of the Graafian vesicles nor any ova or ovula sufficiently far advanced to become separated; but seminal fluid was discovered within the uterus, and the corresponding (?) Fallopian tube, and on the surface of the ovary itself.

Another result of my researches is that, if animals in *heat* be prevented from copulating, the very same phenomena take place in the ovaries as if they had copulated: the Graafian vesicles swell, the ova exhibit all the marks of maturation as if ready to quit the ovary, the vesicle of *Purkinge* disappears under, or is concealed by, an effused spot of blood, and a distinct *corpus luteum* is formed. I have not yet determined with certainty whether the Graafian vesicles burst, and the ova enter the oviduct, or whether these become absorbed under the ecchymosed spot, in the interior of the Graafian vesicles.

That the seminal fluid is ejaculated fairly into the cavity of the uterus—at least in the dog tribe—has been repeatedly ascertained beyond a doubt. In one very interesting experiment, I found in a bitch, immediately after its first copulation, the semen in the superior angle of both cornua of the uterus; while, at the same time, not only were the Graafian vesicles burst, and distinct *Corpora lutea* formed, but no fewer than five ova were found in the left oviduct. It appears therefore indisputable that ova may become detached from the ovary in mammiferous animals before they have ever copulated, and may pass into the oviduct, there to be afterwards fecundated by the seminal fluid.

We cannot, indeed, admit that the ova are detached from the ovary at the time of copulation; for it seems not probable that they could pass so quickly along the Fallopian tubes, as this supposition would imply. Many experiments have led me to believe that it requires fully eight days for them to traverse the whole extent of the oviduct—13 to 16 millimetres long.

But, it may be asked, how does this assertion tally with the results of other experiments, where I have discovered that, in from 18 to 24 hours after the first act of copulation, the Graafian vesicles remained still closed, with the ova included within them; and that the seminal fluid of the male had entered the Fallopian tubes, and even reached the ovaries. Now all this is readily explicable, if it be borne in mind that the act of copulation does not itself occasion any escape of ova from the ovary. The truth is, that, when animals are in *heat*, the ova become maturated and detached from the ovary; and it is well known that it is during this time that the sexual desire impels them to copulate. It is probable, indeed, that, in the natural state, animals gratify the passion almost always before the escape of any ova from the ovaries has taken place, and then the seminal fluid has time to reach the ovaries beforehand. But, if copulation be retarded or altogether prevented, the ova nevertheless pursue their course.

I wlsh now to say a few words respecting a subject that has been much discussed in human physiology, the nature of the *Corpora lutea* in the ovaries of women: Are they, or are they not, to be regarded as signs or evidences of anterior conception? The recent researches of some excellent observers certainly tend to prove that these bodies may be formed, quite independently of sexual connexion. Hence certain authors, as Drs. Montgomery, Lee, Paterson, and others, have tried to shew that there are two sets of the *Corpora lutea,* the true and the false. But the marks of distinction, mentioned by these gentlemen, are quite fallacious; for they are based on mistaken views as to the formation of these bodies.

The old doctrine—that each catamenial discharge depends on a rupture of a Graafian vesicle, the ruptured spot becoming the seat of a Corpus luteum—has been revived of late years; and my own observations tend to confirm the correctness of this opinion; for, on four occasions, where I had the opportunity of examining the ovaries in young women who had died very shortly after the catamenia had been upon them, I found in each case recently-formed *Corpora lutea,* which were manifestly the result of effused blood in the interior of Graafian vesicles.

Now, if we admit this view of the physiology of the menstrual discharge, it is obvious that an intimate analogy must exist between this function in the human female, and the period of *heat* or *rutting* in the lower animals: both depending upon a periodic excitation of the organs of generation, which is connected with the swelling of a Graafian vesicle, and the maturation and escape of an ovum. It is a well-known fact that women conceive most readily immediately after a catamenial period, and it is only when the *heat* has continued for some time, and has reached to a certain degree or pitch in animals, that the desire of sexual connexion is strongly felt. While the discharge continues in the one, and when the *heat* is first felt in the other, there is rather an aversion from, than a longing for, such intercourse. In both classes, the period is co-existent with the maturation of an ovum, and its consequent escape from the ovary.

I hope, ere long, it may be satisfactorily proved that, throughout the whole animal kingdom, (including the human species,) the maturation and detachment of ova from the ovaries obey a certain periodicity—which is manifested in women by the appearance of the menstrual discharge, and in brutes by the period of their *heat:* copulation and fecundation being, in this general point of view, only accidental circumstances.

If the ova of mammiferous animals were not so minute, there is little doubt but that physiologists would often have discovered unfecundated ova in the oviduct, just as we do in all the tribes of birds. But the ova of the former are so small and so delicate that they usually become dissolved in their passage through the genital organs."—*L'Experience.*

---

### AMENORRHŒA, THE CAUSE OF, IN THE STATE OF THE OVARIES.

The following ingenious remarks by M. *Raciborski* will be read with interest after the preceding letter of M. *Bischoff,* as they tend to give a practical value to the speculations therein contained.

" All diseases, which in any way retard the mature and perfect evolution of the Graafian vesicles in the ovaries, will have a tendency to prevent the first appearance of the catamenial discharge at the period of puberty. The best emmenagogue in such cases is the improvement of the general health, and the strengthening of the powers of the constitution. Regular exercise out of doors, wholesome nutritious food, riding, walking, and the various gymnastic games—these are the most useful means to be adopted to promote the efforts of nature to bring forward the development of the ovarian vesicles."

By taking this view of the case, we can readily understand why the first occurrence of the catamenial discharge, after any severe illness, is always to be regarded as a favourable symptom; for it shews that the system is recovering its normal energies in the production of new formations. Hence, too, it is that the period of convalescence is usually fairly established, before the menstrual secretion is re-established.

This is not to be regarded as a crisis, or as in any way contributing to the recovery; it is only as a sign or proof of this having fairly commenced: in short, it is the *effect*, and not the *cause*, of the improved health.

It is important for medical men to attend to this circumstance, with the view of avoiding those errors in practice which have hitherto been too commonly committed. If the catamenial flux be the consequence of a preceding change that has taken place in one of the ovaries, it must be quite obvious that any attempt to force the secretion in a female, who is recovering from a severe illness, before nature has prepared the necessary preliminary process, must be at once futile and pernicious. If the Graafian vesicles are not duly developed, and if, therefore, there be no tendency to a " ponte "—or discharge of a vesicle or ovule from one of the ovaries—taking place, in vain shall we have recourse to the use of emmenagogue medicines, the application of leeches, or the employment of any stimulating injections, &c. The only judicious plan is to fortify the general health and powers of the constitution by a proper hygienic regimen, so as to assist nature in the formative work that is continually going on, at regular intervals, in the female system. In this way alone can we promote the recurrence of the menstrual flow, under the circumstances to which we have been alluding.

*Chlorosis.*—There has been no little discrepancy of opinion respecting the primary or exciting cause of this very common malady. That there exists an alteration or abnormal condition of the blood, in all cases, is abundantly obvious; but then the question presents itself for our consideration—is this state of the circulating fluids primary and idiopathic, or is it consecutive to, and dependent upon, some other abnormal condition of the system?

It has been supposed, by some, that the first link in the morbid change is a lesion of the great sympathetic nerve, and that the alteration in the composition of the blood, as well as the various disorders of the digestive, nutritive, and genital functions, are all owing to a neurosis of this great regulator of organic life. Whatever opinion we adopt on this subject, it will be admitted by all that there is an intimate relation between the condition of the menstrual function and the existence of the disease, to which we give the appellation of Chlorosis, and that this morbid state is one of the most frequent causes of the retardation of its first development in young girls.

The recent researches of MM. *Andral* and *Gavarret* have demonstrated, in a very striking manner, the nature of the remarkable changes in the blood of chlorotic patients: the proportion of the red globules to the other constituents of this fluid becomes diminished by one-half or even two-thirds below the healthy standard.

Of late years, much attention has been paid to the abnormal sounds heard over the heart in most chlorotic cases. There is usually a strong blowing *bruit* accompanying the first note of the tic-tac, that is perceptible over the position of the aortic orifice: it is but feeble and indistinct at other points of the precordial region. With due care on the part of the physician, this auscultatory symptom can never be mistaken for a sign of idiopathic cardiac disease; for the abnormal blowing sound in chlorotic patients is usually of a softer and less grating character; and moreover there is not the extended dulness on percussion, nor the purring sensation, nor the bulging-out of the ribs—so generally present in organic lesions of the heart. Besides the blowing sound heard over the aortic orifice, there is very often a curious sort of musical whistling sound perceptible along the *trajet* of the carotid, and occasionally, also, of the subclavian, and even of the femoral, arteries: this has been very expressively called, by M. *Bouillaud*, the *bruit de diable*—from its resemblance to the sound given out by the plaything of children, which goes by that name.

In addition to the palpitations of the heart, the shortness of breath upon any exertion, the general weakness, the loss of appetite, the desire for inedible, and sometimes even the most offensive, substances—for girls have been known actually to devour their excrements—the constipation, &c., there is a remarkable tendency in chlorotic cases to some form of neuralgic suffering, such as megrims, tic doloureux of the face, gastrodynia, hysteralgia, and so forth.

From the very frequent absence, or, at least great irregularity, of the menstrual secretion in chlorosis, many excellent writers, including *Cullen*, have regarded these two states as being in the relation of cause and effect to each other. Although this opinion is still held by several physicians, it will be found, we think, altogether untenable, upon examining certain cases of the disease occurring in patients who have already menstruated, and in whom all its characteristic symptoms are occasionally developed even before the interruption of the catamenia has taken place—the amenorrhœa, therefore, being consecutive to, and certainly not the occasion of, the chlorotic affection. Indeed, the menstrual secretion is seldom quite arrested, under such circumstances, till the latter disease has continued for a length of time, or exists in a very intense degree. For a period, the discharge may recur regularly, although it is usually much paler and more watery than in health; and it is only when the general atony and feebleness of the system have become so great that (as we may suppose) the ovaries have lost their power of fully developing the Graafian vesicles, that a complete suspension of the menstrual flux occurs. The same reasoning applies to the case of young girls at the period of puberty. If they do not become regular, it is because they are chlorotic; but the reverse of this position does not hold good. In short, the chlorosis is the cause, and not the effect, of the menstrual irregularity.

We have twice had an opportunity of examining the ovaries in chlorotic girls—the one 16, and the other 17, years of age—who died from acute disease, without having ever menstruated. The Graafian vesicles in these cases were decidedly more swollen than they usually are, and ought to be, at that period of life.

The general feebleness of the vital energies of the system at large is, therefore, to be regarded as the real and essential cause of Amenorrhœa in early life. This feebleness is not confined to one part, or one function of the body only; but it affects the entire system, and most probably it is to be traced to the same cause as that which induces the weakness of the digestive organs, and the impoverishment of the blood. We do not, therefore, agree with *Cabanis*, in believing that the proximate cause of Chlorosis is an *inertia* of the generative organs, and the consequent defective or irregular re-action of those organs on the powers of the constitution in general. If such were really the case, and if a certain degree of energy in those organs were necessary to the normal condition of digestion and sanguification, what, we might ask, should be the result of removing the essential organs of re-production—the testicles and ovaries—on the state of these two functions?

The daily experience of farming-life can answer this question most unhesitatingly in the negative. Castration in the lower animals is well-known to be anything but unfavourable to their having a healthy digestion, and a rich state of blood: nay, it seems to promote the activity of these very functions, viz. nutrition and sanguification.

Strange to say, some writers of the present day have supposed that Chlorosis is the result of congestion or engorgement of the uterus, re-acting on the various functions of the economy. "This idea is so utterly groundless, that I should not even have alluded to it," says M. *Raciborski,* "if it were not advocated in a book that ranks high in public favour, and is the production of one who enjoys the greatest reputation for skill in the treatment of uterine affections."* He goes on to state that, when he was chief clinical clerk at La Charité Hospital, scarcely a week passed over without some poor exsanguine chlorotic patients —who had been drained of the little good blood that was in their bodies by leeches and bleedings—being admitted. All this was the effect of the truly absurd notion, that engorgement of the uterus has anything to do with the green-sickness of females.

From what we have said above, it will be obvious that, according to our views, the condition of the genital organs affords no useful indication for directing the treatment of chlorotic affections. The functions of these, as of other, parts of the body, will spontaneously return to a healthy state, when the general powers of the system are restored to their normal activity.—*L'Experience.*

---

### M. Raciborski on the Critical Age in Women.

After some curious observations on the very different periods of life, at which the catamenial secretion has been known to cease, our author proceeds to remark:—

"We are not to regard all Sanguineous discharges from the Vagina, about the *change in life,* as strictly menstrual. Whenever these discharges are irregular in their recurrence, we have strong reason to suspect that they are not connected with the development of the Graafian vesicles, but that they are probably the result of a habit, so to speak, on the part of the system to suffer a certain loss of blood at particular times.

"The cessation, therefore of the Menstrual Secretion is to be regarded in two distinct points of view—1, as indicative of the extinction of the process of ovulation (ponte), and therefore of the faculty of reproduction; and—2, as the arrest of a long-accustomed habit to the periodic discharge of blood, in more or less considerable quantities.

"The physiological extinction of the faculty of reproduction gives rise to effects in every respect similar to those, which have been observed in cases where the ovaries have been removed. All the other functions of the system, and more particularly that of nutrition, seem to have their energies increased; hence the tendency to plethora and corpulency, so common at this period of life. The ill-effects of the cessation of the accustomed discharge are often, as we might expect, aggravated when the woman becomes fat; as she is then generally the less disposed to take active bodily exercise, and the more inclined to indulge in luxurious living.

---

* Although our author does not mention the name of the writer in question, we have no doubt as to whom he alludes. We are pleased to find that the opinion of so intelligent a practitioner as M. *Raciborski* altogether coincides with that we expressed of the Clinique Chirurgicale, in our review of its second volume: vide Medico-Chirurgical Review, for April, 1843.

"The Sexual organs do not in general lose, all of a sudden, the tendency to the periodic excitement of which they have so long been the seat. On the contrary, it usually happens that they continue for some time to be the centre of a fluxion, which terminates at least in a leucorrheal exhalation. *Fernal* relates a case of a woman who, upon the cessation of her Catamenia, was affected every month with an enlargement of the abdomen, which subsided only after a copious evacuation of serous fluid from the vagina.

"Not unfrequently the system, having been for many successive years accustomed to a periodic discharge of blood, seems to experience the want of such a drain after the climacteric period. A state of plethora is induced; and hence a tendency to hæmorrhage or congestion in certain parts is often manifested. *Hoffman* quotes a case, where a woman, soon after the cessation of her Catamenia, was suddenly seized with an apoplectic fit, which did not give way, until a copious hæmorrhage from the hæmorrhoidal vessels took place. Whenever, therefore, there is any tendency to bleeding piles, or to Hæmaturia, at this period of life, the greatest caution should be used in checking these vicarious discharges.

"Certain diseases are more apt to be developed in the female system at the critical age, than at any other period of life. *Hippocrates* says that Gout is apt to make its appearance then—*Mulier podagrâ non laborat, nisi menstrua defuerint.* The correctness, however, of this remark is questionable. *Galen* tells us that he did not find it hold good at Rome; and *Seneca* formally reproaches the women of his time for falsifying the aphorism of the Coan sage.

"However this may be, there can be no difference of opinion respecting the greater frequency of another, and a more distressing, malady at this period of life—we mean Cancer. The old Grecian has not forgot to allude to this: Conclusi uteri menses ad mammas recurrunt, et in mammis tubercula dura exoriuntur; quædam quidem majora, quædam verò minora; hæc autem minimè suppurant, sed semper duriora fiunt; exinde Cancri occulti nascuntur.

"Polypi of the uterus, also, are of not unfrequent development at the critical age. Probably, however, it has been too commonly supposed that this period of life is exposed to unusual danger. It should not be forgotten that many women enjoy much better health after the menses have left them, than they had done for many years before. Perhaps this is most frequently experienced by delicate and weak-blooded subjects, in whom the discharge had been apt to be profuse.

"Women, who have been addicted to venereal excesses, are more subject to suffer at the critical age than others. To such we may apply the poet's remark,

Læta venire Venus, tristis abire solet.

"*Muret* says that the epoch between the 40th and 50th years of female life is not attended with greater danger than that between the 20th and 30th years; and M. *Saucerotte* has shewn, by numerous statistical tables, that the mortality is actually higher between the 30th and 40th, than between the 40th and 50th, years of a woman's life.

"Lastly, M. *Chateauneuf* has proved that the epoch of life between the 40th and 50th years is really more critical for men than for women."

With respect to the medical management of women, in whom the catamenial discharge is gradually ceasing, M. *Raciborski* very judiciously remarks, that more benefit will usually be obtained by regulating the diet and general regime, than by the employment of any pharmaceutic preparations. Whenever there is any tendency to plethora, it is wise to have recourse to a more spare diet than usual, and especially to avoid all stimulating beverages. The occasional use of warm bathing, also, is often of very marked benefit; for thus a greater determination to the surface is promoted, and the more abundant perspiration tends to relieve the system very much.* For the same reason, the woman should accustom herself to take regular active exercise: none is so good as brisk walking for a considerable distance at a time.

M. *Raciborski* is of opinion that not a few of the uterine complaints, that are apt to supervene after the cessation of the Catamenia, are owing to the continuance of sexual intercourse, when nature has intended otherwise. The reproductive faculty no longer exists; and therefore the venereal desire, if such continues to be experienced, is the result rather of a depraved appetite than of a natural impulse. The excitement too, induced thereby in the organs of generation, has no relief now in the periodic hæmorrhage to which they were before subject. The frequency of engorgement of the uterus, under such circumstances, may be readily accounted for; and the subject therefore deserves the serious attention of medical men.—*L'Experience.*

---

### On the Increasing Frequency of Cancerous Diseases.

M. *Tanchou* has recently addressed to the Academy of Sciences an elaborate memoir on this subject. Under the general term of Cancerous Disease, he includes not only the genuine or ulcerated cancer, but also scirrhus, carcinoma, osteo-sarcoma, encephaloid tumors, lupus, noli me tangere, sarcocele, &c.

"The frequency of diseases," says he, "is in the direct ratio of the susceptibility of the organs which become affected. When this is not the case, we may generally trace them either to accidents, or to the agency of some casual circumstances. Cancerous maladies are not exempt from the operation of this general law; but hitherto certainly no satisfactory examination has been made of the causes which usually induce them. I had long suspected that the peculiarities of civilized life may have something to do with the production of this most formidable class of diseases. With the view of determining this point, I have examined with great care the civic registers of the department of the Seine during the ten successive years from 1830 to 1840 inclusive. During these eleven years, the whole mortality in Paris (in the two sub-prefectures of Sceaux and St. Denis) amounted to 382,851 deaths. Of these, 194,735 occurred in males, and 188,116 in females. Of the entire number, 9118 deaths were attributed to cancerous disease; 2163 occurring in men, and 6955 in women. The following is the annual register of deaths from this cause.

| | |
|---|---|
| "In 1830 there were | 568 deaths. |
| 1831 . . . | 865 |
| 1832 . . . | 814 |
| 1833 . . . | 814 |
| 1834 . . . | 857 |
| 1835 . . . | 906 |
| 1836 . . . | 837 |
| 1837 . . . | 778 |
| 1838 . . . | 803 |
| 1839 . . . | 887 |
| 1840 . . . | 889 |
| Total . . | 9118 |

---

* The use of warm baths is unquestionably not sufficiently adopted in this country for the treatment of uterine ailments, whether these occur in early years, or about the change of life. A vast number of cases of Dysmenorrhœa will be more benefited by this simple and agreeable remedy, than by all the other prescriptions of the physician.—*Rev.*

"The class of diseases, to which we are now alluding, is of much greater frequency within the walls, than in the outskirts, of the metropolis. Let us now briefly consider the periods of life at which they occur most frequently. The following table will enable us to form an opinion on this question.

| Age. | Deaths from Cancer. | Males. | Females. |
|---|---|---|---|
| From 1 to 10 years | 23 | 9 | 14 |
| —— 10 to 20 " | 26 | 13 | 13 |
| —— 20 to 30 " | 231 | 62 | 169 |
| —— 30 to 40 " | 1012 | 190 | 822 |
| —— 40 to 50 " | 1975 | 339 | 1636 |
| —— 50 to 60 " | 2108 | 488 | 1620 |
| —— 60 to 70 " | 2067 | 598 | 1469 |
| —— 70 to 80 " | 1315 | 398 | 917 |
| —— 80 to 90 " | 335 | 62 | 273 |
| —— 90 to 100 " | 26 | 4 | 22 |
| Total | 9118 | 2163 | 6955 |

"Of the organs of the body affected, the uterus was most frequently the seat of the malady; next the stomach, and then the mammæ. The liver too is a common seat of malignant degeneration."

M. *Tanchon* remarks, "that there is reason to believe that the frequency of Cancer is on the increase, more especially in large towns. Mr. *Farr* tells us that, in 1838, the number of deaths (in England, we believe) from this cause was 2448, while in 1839 it rose to 2691. My own enquiries lead to a similar conclusion; for, from a registry of cases of Cancer of the Uterus, I find that there were in

| | Cases. | | Cases. |
|---|---|---|---|
| 1830 | 351 | 1833 | 498 |
| 1831 | 391 | 1834 | 436 |
| 1832 | 396 | 1835 | 508 |

"Cancer, like Insanity, is of much more frequent occurrence in civilized than in barbarous countries. Long ago it was remarked in the East, that the disease was much oftener found in the Christian than in the Moslem population there. *Fabricius Hildanus* was of opinion that it was more frequent in temperate than in very warm climates; and this opinion has certainly been confirmed by the experience of some recent observers. M. *Hamon*, an eminent veterinary surgeon who has been attached to Mehemet Ali's service for the last 14 years, informs me that he has never seen a case of genuine cancer in the native or Fellah women, and not many even in the Turkish women, of Egypt. M. *Clot-Bey* has made a similar remark; and M. *Bac*, Surgeon-Major of the Second Regiment of African *Chasseurs*, never met with a single case during the six years that he resided at Senegal. Again, M. *Baudens*, now the head surgeon at the Val de Grace, whose practice at Algiers was most extensive for a considerable number of years, did not meet with more than two or three instances altogether."

M. *Tanchon* closes his remarks with the following general conclusions:

"1. The frequency of cancerous diseases appears to be on the increase, and this frequency seems to be proportionate to the degree of civilization to which a people has attained.

"2. It is towards the decline of life, and more especially in the female sex, that the development of Cancer is most to be dreaded: but early age is not entirely exempt from its attacks.

"3. Glandular organs, and those in particular which are the most susceptible of excitement, are most frequently the seat of the disease.

"4. The proximate cause of the disease appears to exist in every part of the system; but rather in the fluids than in the solids.

"5. When not induced by any external cause, it seems to result from a molecular organic modification or lesion of the part, occasioned by various causes, which as yet are but little known.

"6. In the actual state of our knowledge, the treatment of Cancer can be little else but empirical, like that of Syphilis, Dartres, &c. Still there is reason to hope that we may yet learn how to arrest, if not actually to extirpate, the morbid process. In many cases, we well know that the disease is remarkably stationary for a great number of years, and indeed that its existence seems not much to interfere with the full prolongation of life. What are the circumstances favourable to inducing such an arrest, we indeed are almost wholly ignorant; but much may be expected from a careful watching of such cases by metropolitan surgeons, and others who have the opportunity of seeing many cases of cancerous maladies. The regulation of the diet is unquestionably of primary importance, and hence every means should be adopted to keep the digestive and assimilative organs in a sound healthy state, in order that there may be a regular supply of wholesome chyle."—*Gazette des Hôpitaux.*

---

### The Physiological Antagonism between the Respiratory and the Biliary Organs. By M. Virey.

"The unity of an organized body is maintained only by the aid of certain antagonisms, which are in a state of equilibrium during health. There is, for example, an antagonism between the cerebral and the genital poles in all animals formed of two cemented lateral halves; and there is an antagonism of duality between the brain and the heart. Physiologists have neglected too much these balancing relations between different organs and systems of parts, although much of the harmony of life depends upon their reciprocal agency.

"In the zoophytes, the annelides, and in the six-footed insects—animals in which the respiratory organs are either aquiferous or aerial tracheæ, dispersed over the entire body—there is no liver, nor indeed, properly speaking, any conglobate (conglomerate?) gland. The digestive, like the respiratory, functions, are performed by a general apparatus, and not by special and distinct organs; that of nutrition by a sort of universal imbibition, and that of respiration by means of a tissue that is permeated with tracheæ.

"But, when the organisation becomes more complicated, as in animals provided with branchiæ, (the Mollusca, Crustacea, and Fishes,) then there must co-exist an apparatus, antagonistic of this aquatic respiration, for elaborating the chyme. Such is the Liver. The complexity of the digestive process is found invariably to increase with the more perfect state of the blood-making organs.

"We see, therefore, that as we rise in the scale of animal organisation, the respiratory apparatus acquires more and more ample development; and that, in proportion as this is the case, so is the development of the biliary organs at the same time. Thus, there is an invariable antagonism, or compensating relation between these two sets of organs; and we now proceed to explain the probable reason why such a relation exists. The *Liver* is the organ that is chiefly engaged in elaborating the chyle; or, in other words, in transforming it into an animalised substance, by purifying it from its coarsest particles. The *Branchiæ* or *Lungs* serve for effecting the second and more advanced elaboration, which consists in freeing the blood from a portion of its carbon, in order that its azotised ingredients may predominate.

"The aquatic or branchial tribes of animals—viz. the Mollusca, Crustacea, and Fishes—respiring the air only by the intervention of water, (or, so to speak, at second hand,) have the venous system predominant, and consequently their blood is cold: the liver is usually very largely developed, generally oily, and is sometimes poisonous. In the pulmonary or air-breathing animals, all the functions are usually more highly developed, and none more so than those of sanguification and innervation.

"There is thus always a sort of compensating equilibrium between the organs of the thorax and those of the abdomen, for the due performance of the actions of life. The lung is the principal *foyer* of the arterial blood, while the liver is that of the dark or venous blood.

"The predominance of the respiratory over the biliary functions tends to support and maintain, by means of a red and highly-oxygenated blood, the various organs of external or animal life, especially those of the muscular and nervous systems; whereas, that of the biliary functions indicates an excess, so to speak, of the merely organic or vegetable life.

"In truth, the Liver broods, as it were, like a great reservoir, over the disoxygenated blood; receiving, with the chyle and the lymph, the reparative elements of the economy, in order to transmit them eventually to the pulmonary elaboration. This blood abounds in the elements of carbon and hydrogen, to form the fat and oil, which abound in such large quantities in fishes and other branchial animals. Such also is that morbid state, to which birds, that are fattened for epicurean purposes, are brought: they are stuffed with food, and kept in a state of complete inaction, usually too in the dark and in a highly-heated atmosphere. Whenever, in short, the respiration is slow and imperfect, the liver seems to have an extra amount of duty to perform; and *vice versâ*. Every thing that excites the respiratory functions, such as the inhalation of sharp cold air, the use of acids, and highly-oxygenated substances, &c. increases the energy of the circulation, and tends to induce inflammatory affections of the lungs; at the same time, the activity of the muscular system and the tension of the nervous system becomes exalted; the person is more capable to undergo violent bodily exertions, and protracted mental application; and, in short, all the phenomena of animal life are performed with unusual vivacity and force. The reverse of all this is found to be the case, when the hepatic functions predominate. By attending to this mutual antagonism of the Lungs and the Liver, the physician may be better enabled to afford relief in the morbid conditions of these most important viscera."—*Gazette Medicale.*

*Remarks.*—The speculations of M. *Virey* are usually characterised rather by their fanciful ingenuity than by their solid truth. The fundamental position in the preceding observations—viz. that there is a mutual, and, as it were, compensating relationship between the Lungs on the one hand, and the Liver on the other—is unquestionably perfectly correct; but then, with what is strictly true, he mixes up so much that is either assumed, or that is positively inaccurate, that the reader is often puzzled to know what he may believe. It may be well, therefore, briefly to state that Comparative Physiology affords very numerous arguments in proof of the position that the development and activity of the respiratory and biliary organs are almost invariably inversely proportionate to each other, throughout the animal kingdom. In Insects, in which the respiratory apparatus has a truly wonderful degree of activity and extension, the Liver is so small, that for long it was entirely overlooked, or mistaken for other organs; while in the Mollusca, in which the respiration is comparatively feeble, the Liver is so large as to constitute a large portion of the entire bulk of the body. In Reptiles and Fishes also, whose respiration and temperature are low, the Liver is comparatively larger than in Birds and the Mammalia, whose breathing is energetic, and whose blood is warm. The large size of the Liver too in the fœtus, compared with that of the Lungs, may be adduced as another example of this alternating and compensating relationship between these two viscera.

From all these data, therefore, it seems probable that, when the nature of the animal requires a high temperature, the carbon and hydrogen of the body are eliminated by the Lungs, these elements being disengaged, while oxygen is at the same time absorbed: and, on the other hand, when the animal heat is low, that this elimination is effected chiefly by the agency of the biliary secretion.—*(Rev.)*

---

### On the Natural or Spontaneous Cure of Phthisis.

M. *Boudet*, the author of a very able work, recently published, on the Cure of Pulmonary Phthisis, has a valuable paper on the same subject in a late number of the *Revue Medicale*. The following extracts will enable the reader to judge of its merits.

" Tuberculous degeneration of the lungs and bronchial ganglia is infinitely more common, and is oftener susceptible of a favourable termination, than most medical men are willing to admit. In very young children, indeed, tubercles in the lungs are certainly of rare occurrence. Of 835 dissections of the bodies of infants, during the first year of life, pulmonary tubercles were found in 13 only —or once in every 64 cases. The frequency, however, of the disease increases very rapidly with the age; for, during the second year, the ratio was found to be as that of 1 to 12: and this progresses, as years advance.

" Having examined in succession, and without selection, the state of the lungs in 197 persons, (of from 2 to 70 years of age,) who died from various diseases or even from casual accidents, I obtained the following results. From two to fifteen years, I found tubercles in three-fourths of the cases. At a somewhat more advanced age, the proportion of tuberculous to non-tuberculous individuals seems to reach its maximum; for of 135 persons, whose ages varied from 15 to 36 years, in no fewer than 116 were tubercles found, either in the lungs themselves or in the bronchial glands; viz. a proportion of six in every seven cases. If such be the case, we may truly say that the presence of tubercles in the respiratory organs is the rule, and their absence is the exception.

" This singular result—a result which, at first sight, seems almost quite incredible—is however readily explicable by the gratifying circumstance of the extreme facility, with which these morbid products cease to be incompatible with health, in consequence of various changes that they are liable to undergo in their intimate composition.

" The spontaneous cure of tubercles in the lungs is effected in several different ways. In some cases the tuberculous deposit becomes isolated from the surrounding pulmonary tissue, by a firm fibrous envelope being formed around it. Again, the density of the tubercles themselves may become increased in one of three ways: either by their becoming so desiccated as to form quite a friable paste; or by their assuming a firm tenacious consistence that is greasy to the touch; or, lastly, by their degenerating into an inorganic calcareous matter.

" Tubercles may also disappear, in consequence of the progressive extension of the black pulmonic deposit, that we so often see around them. Occasionally, too, they become wholly or partially absorbed, leaving nothing in their place but their sac or envelope. Lastly, their contents may be eliminated from the body."

These various modes of natural cure may be reduced to five, viz.—1. *Sequestration*, by the development of a fibrous sac around the tuberculous deposit;—2. *Induration*;—3. *Transformation* into black pulmonary matter;—4. *Absorption;* and 5. *Elimination.*

The author makes the following remarks on the latter two modes; and first of absorption.

"Tuberculous matter may be absorbed. I have frequently had occasion to observe tubercles which had become modified in their consistence, and which exhibited very unusual appearances. Instead of being globular, they were of an oval or elliptic shape, or they had become rough and angular on their sides. May we not suppose that such changes were owing to an unequal absorption of different parts of these deposits?

"Occasionally, too, I have found, in the centre of a thin membranous cyst, a minute tubercle, perhaps not larger than the quarter of the size of a millet seed, and which yet exhibited all the physical characters of this morbid product. Now we rarely or never meet with tubercles, when first deposited in the pulmonary parenchyma, so very small as those which we have now described. There is strong reason, therefore, for supposing that a partial absorption has taken place. What greatly confirms the probability of this idea is, that I have occasionally found, in the neighbourhood of these dwarfed tubercles, numerous minute cysts, which were entirely empty; the tuberculous matter, which had once filled them, having disappeared. From these facts I infer that tuberculous deposits may disappear from the tissue of the lungs, by becoming absorbed.

"With respect to the mode by elimination, the only remark that I have to make is, that I have never known it to be effected except in one way, viz. that of expectoration from the bronchi. In this manner, sometimes pieces of very considerable size have been rejected by coughing.

"The transformation of tuberculous matter may take place at all the stages of its evolution; in the state of softening, as well as of crudity; and under the form of grey granulations, and yellow tubercles, whether these be separate or aggregated together.

"Even tuberculous excavations of the lungs not unfrequently undergo a curative process. Of 197 cases taken at hazard, in 10 I have found the cicatrices of caverns in the lungs, without the existence of any recent tubercles; and in other eight cases, the process of cicatrisation was going on, while recently-formed tubercles existed at the same time. When circumstances are favourable, the process of their healing is usually by the organisation of an accidental mucous membrane, lining their cavity; but sometimes by the formation of a fibrous or fibro-cartilaginous envelope. Their cavities may continue to be open, and to communicate, or not, with the adjoining branch. Sometimes they become quite obliterated by the cohesion of their opposite surfaces.

"Usually, the parenchyma of the lung for some little extent around the cicatrised vomica is more or less indurated and impermeable to air: very often it is infiltrated with a black-coloured matter.

"Not only have I frequently ascertained by dissection the frequent transformation of tuberculous deposits, but I have also been able to follow out, in the living subject, the confirmation of these data; and I now feel confident that phthisis is much more frequently cured than most physicians are willing to admit."

M. *Fournet* alludes to his having met with, in the course of one year, no fewer than 14 cases of confirmed phthisis that were cured; besides 10 other cases, in which dissection revealed the traces of caverns, that had become perfectly healed.

He goes on to remark, that "these 14 cases of phthisis, cured in the living subject, have proved to me—

"1. That certain persons, who have exhibited the most decided symptoms of the disease in its most advanced stage, may yet be restored to excellent health.

"2. That, if the general state is satisfactory in these individuals, and does not occasionally bear the evidence in some manner of the accidents of their past life, the local condition is very different, and always reveals the presence of alterations, more or less extensive.

"3. That even hereditary phthisis, in its most advanced stage, is susceptible of cure; although such an occurrence is certainly much more rare than in cases of the accidental disease.

"4. That phthisical patients, who have been treated by very various kinds of remedies, or who have been left entirely to the resources of the natural powers of their economy, seem to have recovered in about the same proportion; and, therefore, that Nature generally 'fait tous les frais' of the cure of the disease."

He concludes his remarks with the following sentence: "The capital fact which seems to spring from these enquiries is, that tuberculous disease is not, like Cancer, essentially incurable; on the contrary, that it is often curable, and that its extreme and most disheartening fatality is referrible rather to the circumstance of its being seated in one of the vital organs of the system, and to its tendency to frequent relapses, than to its primary and essential nature."

*Remarks.*—Would that we could conscientiously take the same views, as to the curability of Pulmonary Consumption, that M. *Fournet* does! Surely he has allowed his enthusiasm on a favourite subject of enquiry to bias his calm and dispassionate judgment; for, in what other way can we explain some of the startling assertions, contained in the preceding observations? Not to say anything as to the alleged extreme frequency of tuberculous disease—and on this point our author is not a little extravagant—we much fear that the experience of other medical men will not warrant the encouraging promises of a cure that M. *Fournet* holds out, even under favourable circumstances; far less in the very advanced stage of the disease, when cavernous excavations have already taken place. We wish not unnecessarily to damp any rational anticipations of effecting a mere *arrest*, if we cannot expect a *cure*, of this very formidable and most distressing disease; but it would be compromising our sincerity and regard for truth, if we hesitated to express, at once and with perfect candour, our opinion as to the degree of influence which such observations as those of M. *Fournet* should be allowed to have.—*Rev.*

---

### M. Cayol on the Vis Medicatrix Naturæ.

The writer of the preceding article having selected for it the following motto—"The ancients attached too much importance to the medicative force of nature in the cure of diseases, while the moderns do not attach enough"—the learned and consistent Editor of the *Revue Medicale* takes the opportunity of appending some remarks in the way of comment.

"This Epigraph," says he, "at the head of an inaugural dissertation, should be received as a happy omen of the decline of the Materialist School of Medicine, and of the return—slow but progressive—of our science to its true principles. It is now 15 or 16 years ago since I commenced, at the Hôpital de la Charité, a course of clinical instruction. My first lecture on the *Vis Medicatrix Naturæ* excited astonishment among the audience that then listened to me. Imbued with the principles of a mere Anatomism—at that time vivified by the genius of *Broussais*, who was then at his apogée—they could form no rational idea of the subject, on which I was discoursing. In the present day, however, the profession is beginning to study and understand it; and altogether a better direction is observable in the anatomical studies of our physicians.

"On the occasion to which we allude, we raised our voice most emphatically against the deplorable abuse that was then made of pathological anatomy, by expecting from it much more than it can ever afford. Physicians were in the habit of holding forth the organic alterations, that were found on dissection, as the causes and 'point de depart' of all diseases; whereas, in most instances, these were the results and effects of the morbid action, that constituted the very elements and essential nature of the disease. Pathological anatomy, we said, instructs us how various structures become gradually changed, degenerated, and perhaps totally disorganised, under the agency of morbific processes, and sometimes by the mere efforts of excessive or over-prolonged re-action—a description of knowledge of the greatest importance in directing our prognosis, and in enabling us to judge aright as to the resources of the healing art. But this is not all. If pathological anatomy teaches us how diseases prove fatal, it also tells us how they sometimes cure antecedent lesions, by revealing to us the admirable resources which the *Vis Medicatrix* of the organism possesses, and the marvellous adaptation of these resources to the end that is to be effected. Thus, in the case of pulmonary excavations, arising from the melting-down of tuberculous deposits, we find that the first act is the exudation of a membraniform lining; which, thin and soft at the beginning, afterwards becomes more and more consistent, and at length is transformed into veritable mucous tissue. While this process is going on, the cavity contracts upon itself; so that, in course of time, perhaps nothing but a mere narrow blind fistula remains. The cure is then as complete as it can be. We have related five cases of this description in our Clinique Medicale: they all occurred, during the year 1824, in the wards of La Charité Hospital."—*Revue Medicale.*

It must indeed be gratifying to an intelligent observer like M. *Cayol* to watch the re-action that has been going on of late years in the tone and prevailing spirit of medical doctrine, more especially in his own country; and to use his own, no inconsiderable, influence in bringing it again under the yolk of calm observation and unprejudiced reasoning. The enthusiasm of the phlogistic revolutionists is unquestionably giving way on every side to the quiet loyalty of the Hippocratic School—of which our author has long been one of the most steady and able supporters.—*Rev.*

---

### THE VEGETABLE ORIGIN OF PORRIGO DECALVANS.

*Porrigo decalvans* is a disease of the skin, usually of the scalp, producing the falling-off of the hair from the parts affected. It is characterised by rounded spots or patches, the surface of which is usually covered with small dry scales, or a white bran-like powder. If this powder be examined with the microscope, it will be found to consist of minute *cryptogamic* plants. The hairs, that have fallen off, are observed to be encrusted on all sides with these singular growths, and to form a veritable vegetable sheath, which invests them from their point of emergence from the skin to the extent of three or four millimetres. M. *Gruby* (the discoverer, we believe, of this pathological curiosity) has denominated the Fungus by the appellation of *microsporon Andouini,* in compliment to M. *Andouin,* whose enquiries have done so much to illustrate the nature of the parasitic plants, which infest the tissues of living animals.

This porriginous Fungus commences its development on the surface of the hairs, at the distance of one or two *millimetres* from the epidermis; the first sign of its existence being that the tissue of the hair is observed to lose its transparency, in consequence of the formation of excessively minute molecules on its surface.

The altered tissue exhibits the appearance of having distinct fibres, and of cells larger than the fibres of the hairs, elongated and situated parallel to their axes. It is in this part that the *microsporon* is first perceived. By gradually extending in all directions, the adjoining hairs are quickly affected in a similar manner, and the morbid growth advances, until patches of the affected hair fall off, and leave the skin nearly quite bald.

These Fungi are developed and multiplied with surprising rapidity; and hence the extension of the diseased patches is sometimes very sudden. The hairs usually break off at the point, where they are invested with the vegetable covering. The thickest hairs resist the invasion of the disease the longest.

The vegetable nature of *Porrigo decalvans* is a fact that speaks for the contagiousness of the disease. How far any of the other varieties of this scalp-affection are traceable to a similar origin, has not yet been ascertained. The first step to the cure of a disease being to understand its nature, we may fairly anticipate that the discovery of M. *Gruby* may have a therapeutic, as well as a physiological, interest.—*Revue Medicale.*

---

### Remarks on Lateral Curvatures of the Spine.

As connected with the subject—alluded to in a preceding article—of the morbid states induced by, or at least associated with, the first appearance of the catamenia in girls, we may here introduce the following very sensible remarks on lateral Curvature of the spine. It is well known that this deformity is tenfold more frequent in the female than in the male sex; hence we are, from this circumstance alone, led to suspect that the origin of the affection may generally be traced to some peculiarity in the constitution of the former. True it is, that the pernicious system of over-education, to which girls are so much more frequently subjected than boys, the want of due exercise in the open air, the prolonged sedentary occupations—often, too, on stools or benches which have no back supports—and the insufficiency of school diet, must powerfully contribute to increase any tendency to weakness in the back—more especially at that period of life when the body is growing the fastest, and when, moreover, the curious and important function of menstruation is beginning to be first established. How different is the system pursued with boys at the same age! They have now usually more out-door occupation than before; they are encouraged to ride, fence, and swim; they begin to feed like their fathers and elder brothers; and, in short, every thing is done to make them hardy and robust, at this epoch of transition from boyhood to puberty.

It is, unquestionably, about the period of the first menstruation that the earliest signs of spinal curvature are observed in a large majority of cases; and we need scarcely add, that it is at this time that judicious treatment can do the most to check the incipient deformity. Parents are too apt to suppose that it is usually to a mechanical injury, such as a fall or blow on the back, that the malady may be traced. Far from it; in the greater number of instances, no direct injury has ever been received, and the evil has resulted, either from a vicious habit of standing or sitting, or from excessive occupation in the upright position.

Curvature of the spine may exist for a long time quite unnoticed by persons who are ignorant of orthopædic studies. To the practised eye, however, of a professional observer, it is usually indicated by some slight irregularity of the shape; as, for example, the greater elevation of one shoulder than of the other, or by some awkwardness in the attitude, manifested by the girl when she is standing or walking. Unfortunately, parents and teachers very often fall into the grievous error of imputing to wilfulness of temper, what is, in truth, the effect of an incipient deformity, and they attempt to rectify the evil by punishing the child, instead of by consulting their medical adviser.

That late most excellent observer, M. *Delpech,* who has, perhaps, done more for the advancement of scientific orthopædy than any other writer, has some good remarks on this subject.

"At the age," says he, "when deformities usually commence, young persons have scarcely any other motive or calculation for their conduct, than that of instinctive impulse. What mere freak of mind, we might ask, would ever lead them to acquire strange and awkward attitudes, which have neither meaning nor convenience? We may rest assured, that such attitudes are the result of some positive fault in the organisation; and hence, although the patients themselves have no idea of its existence, it is the duty of those, who have the charge of them, to find it out. Nothing is more blameable than the too common practice of punishing young persons under such circumstances; for their will has, in reality, nothing to do with the awkwardness of their attitudes.

"The weights, with which certain parts of the dress are loaded, and the straps and buckles, &c. that are vainly used to prevent the unseemliness of shape or position, are all more or less directly injurious: such means can never be of any utility; and fortunate it will be if they do not prove to be seriously hurtful. In truth, the very attitudes, of which complaint is made, should be regarded as emphatic symptoms, or palpable indications, that require to be attentively studied and examined by the medical man, and a just appreciation of which, at a sufficiently early period, may serve to suggest the proper hygienic measures to be adopted."

It is by watching the manner in which a young person stands, and her attitude when she sits or gives her hand to another person, and so forth, that we may often detect the very commencement of the infirmity in question. For example, it is often observable that, when there is any deviation of the spine, the girl seldom places both her feet, while standing, in the same line; one is usually more advanced than the other. This is owing to the increased (relatively) length of the limb, on the side on which the concavity of the curvature exists.

Again, the girl will generally prefer to sit on a low-backed chair, so that she may be able to put her arms over its back, and thus give more support to the weakened spine. In walking too with another person, she will very generally give that arm which corresponds with the concavity of the curvature; and this, we need scarcely add, is almost always the left one; seeing that, in a very large majority of cases, the curvature of the spine leans with its convexity to the right side. A not unfrequent indicative symptom of incipient spinal curvature is a dull uneasiness or pain on one side of the chest, sometimes immediately under one breast, or perhaps in the epigastrium: although very seldom severe, it may vary a good deal in intensity at different times.

The usual method of examining the back, for the purpose of detecting spinal irregularities, is to judge by the line formed by the spinous processes of the vertebræ. By trusting, however, exclusively to the information thus obtained, the medical man is apt to fall into a mistake, as the deviation may have advanced to a considerable extent, before any alteration in the direction of these processes is at all perceptible. M. *Guerin* has quite satisfactorily shewn that spinal curvatures arise, in the majority of instances, from a veritable twisting of the anterior parts or bodies of the vertebræ, the posterior parts or the rings and the spinous apophyses being but little affected, until the deformity has made more progress. We should therefore remember that the dorsal spine may appear to the eye perfectly straight, while the transverse processes, which participate in the torsion of the bodies of the vertebræ, already indicate the state of the column by the peculiar appearance of the ribs and the deep-seated muscles, which lie in the vertebral hollows. The ribs, being fixed to the extremities of these (the transverse) processes, necessarily experience a displacement backwards, when they are twisted by following the direction of the bodies of the vertebræ. Hence arises the projection of the vertebral hollow, along the whole extent of the deviation. This abnormal projection always corresponds with the convex side of the column. In part, it is real and absolute; and, in part, it is relative or owing to the depression of the hollow on the other side of the vertebræ, produced by the dragging forwards of the transverse processes and the ribs on this side.

*The abnormal prominence of one of the vertebral hollows (gouttiers) may therefore be regarded as a fundamental character of incipient curvature of the spine.* The seat and degree of this prominence are always proportionate to the extent of the irregularity. It is usually most perceptible in the upper part of the back, in consequence of the scapula there increasing the projection of the ribs, and thus rendering the deformity more apparent. This is the reason of the circumstance that most parents notice the awkward elevation of one of the shoulders, before the back is even suspected to be the seat of the evil.

At first, only a single deviation is usually discoverable; but, in course of time, one or more others are established: these may be called balancing or counterpoising curvatures. They are always in the opposite direction from the primary one; and seem to be induced by Nature, to enable the trunk to recover its original centre of gravity, which had been deranged by the first deviation. Hence it happens that, in many cases, we may observe several alternate projections and depressions along the line of the vertebral column.—*l'Experience.*

---

### M. Pouchet on Fecundation in Mammiferous Animals.

The phenomena of generation in the human species follow certain simple laws, which are perfectly analogous with those which prevail throughout the whole animal kingdom, except among the very lowest members of the scale. Such is the opinion of our author, who, in this respect, seeks to establish the operation of a much more comprehensive principle than either M. *Duvernay* or M. *Bischoff*—his talented rivals in this interesting field of enquiry—have yet ventured to enunciate. These two last-named gentlemen admit the entire conformity of the phenomena of generation in the different families of the Vertebrated family; but they do not attempt to trace the analogy among the Invertebrata. Whether M. *Pouchet* is strictly correct in all his views, can be determined only by the results of future researches; but all must admit that his recently published work entitled—Theorie positive de la Fecundation des Mammiferes basée sur l'Observation de toute la serie Animale; Paris, 1843—deserves the highest praise for its ingenuity and talent.

He is of opinion that in all animals—except perhaps in the very lowest groupes—the fundamental phenomena of generation consist—1, in the production of a certain number of ovula or eggs, contained within an organ called an ovarium, and—2, in the subsequent fecundation of these ovula by a special fluid secreted by the male organs.

The ovula are expelled from the ovaria at regular determinate periods or epochs; but they never become developed, nor produce living germs, until they have been brought into contact with the seminal fluid. If this contact be wanting, they become decomposed and cease to exist.

The contact of the vivifying fluid takes place in a special part appointed for the purpose, although its relative situation to that of the ovarium varies much in different animals. It may however be asserted, as a general principle or law, that the ova must have attained to a certain degree of development and been discharged from their lodgement in the ovary, before the action of the seminal fluid can take place effectually. It is usually during its *trajet* along the sexual canal, that the ovum becomes fecundated. Very often, however, this act takes place entirely out of the body of the female; as, for example, in fishes and reptiles. When this is the case, we may suppose that, even in the ovary, the reproducing bodies or ova have not yet attained that degree of development, which is necessary before they can receive the vivifying impression: often, too, the seminal fluid could not reach so far as might be requisite to impregnate them.

No doubt can reasonably be held as to the identity of the generative process in all truly oviparous animals. The extreme smallness of the ova, when first discharged from the ovaries, in certain viviparous members of the vertebrated class, caused them to be long entirely overlooked; and, indeed, it is only of late years that they have been satisfactorily seen and examined. Hence arose the uncertainty of opinion as to the mode in which generation was effected in these animals; and hence the reason that it was believed by many physiologists that the process was quite distinct from that followed in oviparous animals. The researches however of modern enquirers have clearly shewn that, in all animals alike, and in the human subject also, the same general law holds good—viz. that ova are invariably formed, and are expelled from an egg-bearing organ, known by the name of ovarium. There is thus a very intimate relationship and affinity between the various tribes of the animal series, however widely these may differ in point of complexity of organisation and variety of outward form.

It is only because the ovum of the human subject was examined when out of the ovarium, and after it had undergone a certain degree of development within the cavity of the uterus, that physiologists were led to regard it as exhibiting important differences from that of other mammals and of birds. But, if it be studied while yet in its formative *nidus*, we cannot fail to perceive that it is in every respect similar to the ovum of these animals, with the exception of its being unusually small: in fundamental structure, and in physiological import, both are entirely alike.

Moreover, observation and experiments have abundantly proved, in the most convincing manner, that the processes of fecundation and of the development of the ovum obey the same laws in the woman, as in the females of the inferior animals.

The ova, when once formed and discharged from the ovary, become developed either within or without the body of the animal, after they have been exposed to the influence of the spermatic fluid; but the conditions, which they must present for this act, are not manifested, until they (the ova) traverse the generative organs. The difference, which exists between oviparous and viviparous animals in this respect, is not so important as might be imagined; for, in truth, there is a gradual, and not any abrupt, transition from the one tribe or family to the other.

In all animals, the ova are discharged at determinate epochs, which have a manifest relation with the periodic excitement of the genital organs. It has never been disputed that, in birds, reptiles, fishes, and in the invertebrata, there is a regular and determinate formation of ova; and, even in the case of mammiferous animals, although the function of the ovaries was never well understood, it was notorious that reproduction generally took place at fixed and regular periods. With respect however to them, and also to birds, it should be borne in mind that fecundation may be repeated much more frequently, when the animals are living in a state of domesticity, than in their natural condition. But we are not to infer from this circumstance, that there is ever a continuous and uninterrupted action in the ovaries. The best laying hen rests during cold weather; and often we find, in the case of our domestic animals, that when we force them as it were to copulation, at unseasonable periods, the act is unfruitful; because it takes place when they are not naturally in *heat*. It is quite a mistake to suppose that the season has nothing to do with the process of reproduction, even in the case of those animals which, from living in a state of domesticity, are so much more frequently rutting, than when they are in a state of nature. We may therefore assert that, in all animals without exception, the escape of ova from the ovaries takes place only at stated intervals.

The period, at which this escape occurs in most mammiferous animals, is indicated by the turgescence and excitation of the genital organs; in some there is a sanguineous discharge from the vagina at the same time. Now the same holds good in the human species. The female is subject to phases of excitation, and phases of intermittence. It is only during the first that the ova are produced, and that fecundation is possible. If the periods, when reproduction is possible, are very frequent in the case of women, this is manifestly owing to the comforts of social life. But even with her, we may follow the trace of these intermittent periods, and determine the epochs of their return with precision, just as we can do in the whole Zoological series.

In mammiferous animals, fecundation never takes place, except when the emission of the ovules coincides with the presence of the seminal fluid; in other words, impregnation can only occur when the ovum, divested of the coverings it had in the ovarium, is carried forwards free into the genital passages, and is there exposed to the action of the vivifying fluid.

The discharge of the catamenia in the human female corresponds with the phenomena of excitation, which the females of all, and more especially of the mammiferous, animals exhibit at their periods of *heat.* In certain tribes of animals there is a red-coloured discharge from the vagina at the same time; in others, this discharge is only a mucous one; but in both instances it is the result of the same cause, viz. the erethism of the internal organs. It is not, therefore, strictly correct to say, that the human female alone is subject to a periodic discharge from the uterus, as a similar or analogous phenomenon takes place in all mammiferous animals at certain determinate epochs. Much depends in this respect on climate, mode of life, &c. In some nations, the menstrual flux in women is scarcely sanguinolent at all; and every now and then, we meet in professional practice with instances where conception readily takes place, and yet the female has never menstruated at all. The identity between menstruation in the human female and the epoch of rutting in the lower animals, being admitted, and as it is only at this epoch that fecundation is possible in the latter, we may fairly infer that menstruation should be regarded as the monthly index of the generative capacity in the former.

Fecundation has an intimate and constant relation with the menstrual flux. It is, therefore, easy, in the case of the human female, to determine with precision the inter-menstrual period when conception is physically impossible, and that when it may probably occur. It is universally admitted that conception takes place most readily about the time, that immediately follows a menstrual period. But certainly this is not always the case; and we have every reason to believe that impregnation may occur three, four, or even seven days, if not upwards, after the secretion has ceased. To understand this aright, we should bear in mind that this act does not always take place at the moment of coition, but may occur some time after, when the ovum, detached from its *nidus* in the ovarium, comes to pass through the organs still moistened with the spermatic fluid.

Properly speaking, there is never such a thing as an ovarian conception. Doubtless, it is possible, in some extraordinary cases, that an ovum, while escaping from its capsule, may be fecundated by the semen which the dilated end of the Fallopian tube may shed upon it; and then, that it may become developed on the surface of the ovarium, forming adhesions and connexions with this organ. But this supposition, it will be observed, is very different from believing that an ovum ever becomes impregnated, while it remains in a Graafian vesicle that is covered with its peritoneal lining.

The views of M. *Pouchet* are in strict accordance, as we have already said, with those professed by M. *Duvernay* and M. *Bischoff*. They all agree on these important points—1, that generation is effected (at least in vertebrated animals) by means of ova which are formed antecedently to conception; 2, that the formation of the ova is quite independent of the action of the seminal fluid; 3, that the process of fecudation, however, requires the ova to be impregnated by this fluid; 4, that the periods of rutting in animals correspond with the periods of menstruation in the human female; and 5, that the resemblance between these periods enables us to form an idea of the probabilities of fecundation, in the various tribes of animals.—*Gazette Medicale.*

---

### Marriage, in a Social and Medical Point of View.

Polygamy, so far from being favourable, as might at first be supposed, to the increase of population, is found by statistical experience to be most baneful, by destroying the appointed equilibrium between the two sexes, and by giving rise to a race that is feeble and puny alike in mind and body. This is a fact abundantly proved by past history, as well as by the actual condition of many nations in the East at the present day.

On the other hand, Celibacy is equally, and more directly, injurious to the interests of society; because, not only is the amount of offspring, that might be expected, thus directly curtailed, but the proportion of deaths is found to be considerably higher among bachelors than among married men.

"If," says M. *Marc,* "we take *four* as about the average number of children born in the married state, it will follow that, in the space of from 25 to 30 years —the ordinary duration of fecundity in the female—a hundred celibitaires have deprived society of at least 360 citizens. Was there ever a war so destructive or so depopulating as to cause such a baneful result to society as this?"

*Montesquieu* long ago remarked that illicit connections contribute but little to the propagation of the species; and the experience of every nation fully bears out the truth of this observation. The excesses, which most unmarried persons indulge in, render such connections very generally unfruitful; and, moreover, of the small number of children, that are the offspring of debauched intercourse, the greater number perish from want of due attention after birth.

Thus nature, as well as religion, clearly shews that reproduction, to be successful on a great scale, must be sought for only in the married state. The laws of every country, and of every age, have alike recognised the truth of this principle. The higher privileges and superior protection, awarded to the offspring of legitimate unions, bear abundant testimony to the fact.

Not a little difference of opinion has existed among moralists and political writers, as well as among medical men, as to the proper age at which marriage should generally be contracted. The politician has looked at the question only in, what may be called, a statistical point of view, as far as it affects the population and powers of subsistence of a country; the moralist, in its relation to the manners and social virtues of the state; while the physician has attended more especially to the influence exercised thereby on the hygiene and physical vigour of the inhabitants.

Most of the economists (as they are called) in the present day, with *Malthus* at their head, have recommended that the period of contracting marriage in both sexes should be considerably retarded. They allege the following reason for this advice.

It is an admitted fact, that the population of a country has a tendency to double itself in about 25 years, and that its increase goes on in a geometrical ratio, while the powers of subsistence, under the most favourable circumstances, can never be made to advance in anything like the same degree. According to the calculations that have been made, 100 acres of the most productive ground, which may be able to subsist, in the present day, a hundred persons, would not be capable of producing, even in the course of 300 years' cultivation, food sufficient for 200 persons; while, in the same period of time, the offspring of these hundred persons might, according to the geometrical ratio of increase, amount to no fewer than 409,600 individuals.

We do not dispute the truth of these statistical statements, and we willingly give credit to the good intentions of M. *Malthus*,—who, it should be remembered, has never pushed the principles of his system to the extravagant, and occasionally also the immoral, extent which some of his followers have done; but we must be permitted to question their soundness even in a mere social point of view.

There has been considerable difference of opinion as to the earliest period of life, at which marriage should be contracted. Some have hastily said that, whenever the catamenial secretion is fairly established, the girl is quite capable of conceiving. Although the occurrence of this peculiar function is unquestionably a sign or indication of the commencement of the reproductive faculty in the female, it does not necessarily follow that the person can at once become a mother of healthy children. *Burdach* very wisely remarks that, "the genuine maturity of the system, to which we give the term of *nubility*, differs very materially from mere puberty. It is quite necessary that an organic power or faculty should exist for some time, before it is called into exercise, in order that it may be fairly and fully developed, and be able to exercise all its designed influence and powers."

Premature sexual connection is as injurious in the case of the human species as of the lower animals, and alike to the parents and to the offspring.

The same law holds good in the vegetable world. Young plants quickly perish, if they begin to bear flowers very early; and dogs and other animals seldom attain their full strength and size, if they are allowed to obey their generative instincts at first. The finest breeds of sheep, horses, cattle and swine are derived from a parent stock that had reached perfect maturity.

---

### Observations on Infanticide. By M. Duvergie.

Infanticide is defined, in the 300th Article of the French Penal Code, to be the Murder of a new-born infant. There is some degree of obscurity in this definition, arising from the circumstance that no reference is made to the question whether the infant, at the time of birth, is *viable* (capable of supporting life) or not.

We must therefore inquire whether it is sufficient that the child, in such a case, be born merely alive or *quick*—or if it is necessary that it be also *viable*.

It is important to attend to this distinction in all considerations of Legal Medicine, which have reference to the crime of Infanticide. On the one hand, a fœtus may possess a most vigorous *vitality;* but, either from being prematurely born, or from some other cause, it may not be capable of living after being separated from the parent; and, on the other hand, it may be perfectly *viable*, and yet it may be still-born, or dead at the period of birth.

In reference to the important question of succession to property, the infant, when born, must, according to our laws, be not only *quick* or alive, but also *viable;* and many of our best jurisconsults have contended for an extension of the same principle in cases of alleged Infanticide.

For example, M. *Rogron*, in his Commentaries on the Penal Code, says: "An indispensable condition to make out a case of Infanticide is that the child was born *viable* (habilis vitæ). The mere circumstance of its having uttered a few cries at the time of birth is not a sufficient proof of this, if the organisation shews that the life, which seemed to animate it, were only 'un souffle passager.' The infant, which when born is not *viable*, is not deemed to have an existence in the eyes of the law; and consequently there cannot be murder of a being, that is not alive."

M. *Duvergie* differs from M. *Rogron* in the interpretation of this question. He is of opinion that, to establish the crime of Infanticide, it is only necessary to shew that the child, when born, was quick or alive—whether it was *viable* at the time, or not.

The question of Vitality has, he thinks, nothing to do with criminal, although it certainly has with civil, cases.

It has been doubted by some writers whether the law respecting Infanticide can be made to apply to those cases, where the infant is born before the full period of gestation: but this is evidently an untenable objection; as it will be observed that the code does not specify or mention any particular period of development, and merely uses the phrase *new-born*. The crime of Infanticide may be committed upon a child of eight, seven, or only six months of intra-uterine life, if it can be proved that, at the time of delivery, it was living, even although it may never have breathed.

M. *Duvergie* quotes a case, on which he was once officially called to report, and in which he gave an opinion that the child had been murdered; although, from the state of the lungs found on dissection, it was evident that respiration had never taken place. The fœtus was between the seventh and eighth months of its age, and was well formed in every part. On examination, a deep cut was observed on the crown of the head; and within, as well as on the outside, of the cranium there were distinct marks of severe contusion.

The lungs at once sunk in water; and, when squeezed under its surface, no bubbles of air were perceived to arise.

It is right that this important question—whether the circumstance of breathing having once taken place is to be considered as necessary to our admission of the life of the child, in cases of alleged infanticide—should be settled. Whatever opinion is come to, it is quite obvious that medical men in particular should ever bear in mind the important difference between *vitality* and *viability*.—*Annales d'Hygiene, &c.*

---

### ON CERTAIN PERIODIC OR INTERMITTENT AFFECTIONS OF THE EYE.

The inflammations of the eye and of its appendages, like a great number of other inflammatory affections, are occasionally observed to exhibit a distinct character of intermittence.

In some cases, the disease returns at distant, although nearly regular, periods, and at certain seasons of the year; while, in other instances, the attacks recur at stated hours every day, or regularly after the lapse of two or three days. It is especially necessary, in a practical point of view, for medical men to be acquainted with the latter description of cases; because, if the intermittent character or type of the disease is not recognised, a depletory treatment may be persevered in for a most unnecessary length of time, to the great prejudice of the patient's general health, and to the decided aggravation of the local malady. The disease is not so unfrequent as many suppose.

Several of the older writers seem to have been well acquainted with intermittent Ophthalmia.

In the works of *Morton, Hoffmann, Van Swieten, Sauvages*, &c. we meet with many very interesting cases of ophthalmia, which, after resisting all antiphlogistic remedies, speedily gave way to the use of anti-periodic medicines. Bark and Arsenic are, as a matter of course, by far the most efficient remedies. The difficulty lies not in the treatment, but in the diagnosis, of the disease. Unless the existence of the intermittent type or character is detected, no local application will be found of permanent advantage. If the inflammation be of recent date, perhaps no Collyrium is better than poppy decoction applied warm; but if it has existed long, a weak solution of Sulphate of Zinc—to which a little Hydrocyanic Acid may be often added with great advantage—will be most expedient.

It deserves to be noticed that, intermittent Ophthalmia is not unfrequently associated with Neuralgia of the supra-orbital nerve, or *Brow-ague*, as it is often called.—*Gazette des Hôpitaux.*

---

### Medical Memoranda.

The following rather fanciful observations are from the pen of M. *Gouraud*, the chief physician of the Invalid Infirmary at Avignon in the South of France.

### *Reciprocal Influence of the Nervous and Sanguiferous Systems.*

The bloodvessel and the nervous fibre are the first parts which receive life, and the last which lose it. Anatomy shews that they are always associated together in the cellular substance, which serves as a bond of union between them; Physiology displays them invariably acting in unison,—and Pathology finds them very generally acting the one upon the other. Let us cite a few examples in illustration of these propositions:

A young girl, returning home one morning, was insulted by a soldier who clasped her round the waist. She chanced to have the catamenia upon her at the time; the secretion was at once checked, and did not again return.

The mother of one of our young soldiers in the army of Italy, 1798, was told in our presence of the death of her son: she started up for a second, and the menstrual discharge ceased that very moment.

These are instances of the action of the nervous on the sanguiferous system: the following exhibit the action of the sanguiferous on the nervous.

A young Creole girl, of an hysterical constitution, was seized with spasm of the throat, which for two days prevented her from swallowing any thing. She was bled; and, from the moment that the blood began to flow, this spasm gave way, and she could swallow with ease.

A plethoric woman is advanced beyond the middle of pregnancy without having quickened; draw a few ounces of blood from her, and the first movements of the fœtus will probably be felt forthwith. * * *

*Of Asphyxia.*—What do we mean by this term?—the accidental privation of all the *signs* of life. This sudden death may be only apparent; all the organs of the body do not usually die at one and the same time. The popular saying, that "the eyes are the mirror of the soul," and the results of various experiments on animals, seem to warrant the belief that the eyes are (probably) susceptible of surviving the other organs, and that a spark of life may remain latent in a nervous filament or a minute bloodvessel of the eye, for a brief time after the cessation of vitality in other parts. *Flourens* tells us that, "when the brain, properly so called, is removed, the animal loses all intelligence; but in reference to the eye nothing is changed. Objects continue to be painted on the retina as perfectly as before, the iris continues to contract, and the optic nerve retains its excitability."

Let the medical practitioner therefore bear in mind, when he is called to a case of asphyxia, which is the nerve and the bloodvessel that are the last to die: let him but breathe on this spark; and he may have the happiness of re-lighting the torch of life: *vitæ lateat scintillula forsan.*

One morning in the Autumn of 1840, I happened to be passing along the Rue de Vaugirard, when I heard a cry of alarm. A man had been just drawn out from a sewer, in a state of complete asphyxia: no pulse was to be felt, and no movements of respiration visible: the body however retained its heat, although in every other respect all signs of life had quite ceased. I at once opened a vein in the arm; the blood spouted out to some distance in a continued stream: still no mark of returning vitality. I then separated the lids of one eye, and jerked a few drops of brandy on its surface: the lids at once contracted upon it. The attendants were delighted; *anima enim ipsius in ipso est*. The application of the brandy was repeated to both eyes; the muscles of the face became retracted; the pulse began to be felt, and at length the man was observed to breathe. A spoonful of cold water was put into his mouth, and he swallowed it. At the end of two hours, ' le mieux fut decisif,' and subsequently he quite recovered.

M. *Gouraud* seems to have but little faith in the employment of artificial respiration in cases of asphyxia. He says, in reference to the case he has just reported, " pulmonary insufflation was not tried! it would have been useless, as the office of the lungs is to maintain personal life, not to restore it when it has been suspended. The relief must therefore proceed from a higher source,—that is to say, from the two first moving powers of life,—the nervous and the sanguiferous systems."

Such reasoning as this, to say the least of it, is certainly not very satisfying. May not an impression be made on the nerves of the air tubes by the insufflation of air along their canals, just in the same manner as an impression is made on the ophthalmic nerves by applying a stimulant to the eye?—*Archives de la Medecine.*

---

### M. DUMAS ON THE FATTENING OF CATTLE.

No subject is occupying more attention in the present day than Animal Chemistry. The researches of *Liebig* in Germany, and of M. *Dumas* and others in France, have given, of late years, a strong impulse to this curious and very important branch of knowledge. The latter philosopher, in conjunction with MM. *Boussaingault* and *Payen*, has recently published a Memoir, the object of which is to ascertain the mechanism, so to speak, of the accumulation of fat in the tissues of the different organs of the body on the one hand, and of the formation of milk in the mammæ on the other.

It has hitherto been the general opinion that all fatty matters—whether in animals or vegetables—were the product of a certain eliminating function or process, that is effected at the expense of the food that is taken into the system. The researches however of these three gentlemen tend to shew that the fatty substance is primarily formed in plants, and that it passes ready formed into the body of the animal; and is then either immediately consumed in the production of animal heat, or else is stored up for a time in different parts of the body, as a magazine of reserve for the respiration at some future time. The main point of their researches is to shew that the fat of animals is not a product of the organization of their own bodies, but is derived solely and ready-prepared from the vegetable world. The fatty degeneration, to which animal substances are subject by slow decomposition, is supposed to be in some degree an argument in favour of this idea. Our authors believe that the *adipocire* is not generated after death, but merely becomes exposed and separated from the fibrine, with which it was previously incorporated, in consequence of the process of slow decay.

Some facts in the physiology of animals appear to countenance this opinion. For example, there is certainly a great difference in the chyle of carnivorous animals, according as they are fed, on the one hand, on vegetable matters which are rich in feculæ or sugar, or on lean meat; or, on the other hand, on food that contains a good deal of fatty matter. In the first case, the chyle is found to be transparent and serous, and it yields very little to æther when mixed with it. On the contrary, in the second case, it is observed to be opaque, of a creamy aspect, very rich in globules, and gives a great deal of oleaginous matter to ether. Now we can follow with perfect accuracy the fatty ingredients of food (converted by the process of digestion into a sort of emulsion) in their passage, first into the chyle and afterwards into the blood—in which it remains for a length of time unaltered, and, so to speak, at the disposal of the system. This progressive transmission of the fatty ingredients of the food, through the alimentary canal into the blood, is believed to take place without their undergoing any change in their physical properties.

This opinion is probably quite correct in reference to Carnivorous Animals; but, if we extend it to the Herbivorous, two difficulties will be found to present themselves to our notice. It may be asked, where can we find in plants a sufficient quantity of fatty matter to account, not only for the production of milk, but also for the fattening, at the same time, of the cattle fed upon them? Is it not more simple, it may be said, to suppose that the butter and fat are produced by some process of transformation of the saccharine matter, which vegetable food is know to contain? In answer to this objection, we may state that the numerous experiments made by M. *Payen* on the grasses and other vegetable substances, on which cattle feed, will serve to explain away most of those difficulties. They have clearly shewn that fatty matter exists very generally in the texture of most plants that serve as food for animals. Though most abundant in the substance of the seed, it is present also, in varying quantities, both in the stem and leaves. In the latter organs, the fatty matter exists usually in the form, and having the characters of wax. This, being received into the stomach of an herbivorous animal, gradually finds its way into the blood; and, by then being exposed to the oxygen of the air during the process of respiration, it becomes partially oxydated and is converted into the *Stearic* and *Oleic* Acid, which we find in tallow. By undergoing a second elaboration in the bodies of Carnivorous Animals, this same matter, being oxydated anew, produces *Margaric* Acid—characteristic of true fat. At length, when still more highly charged with oxygen, the volatile fatty acids of animal bodies, such as we find to be present in the blood and perspiration, are generated. It is always to be remembered that these substances may be converted, by a process of slow Combustion, into Carbonic Acid and water, and be thus eliminated from the system.

Independently of the fatty matter which herbivorous animals derive, ready formed and in a direct manner, from the plants which they feed upon, the researches of M. *Dumas* have shewn that one of the principles of sugar—the olefiant gas—may (after various transformations) have something to do with the formation of fat in their bodies; and, therefore, that part of the adipose deposits of these animals may perhaps be thus accounted for. But this is unquestionably a very inferior and subordinate source of their supply; and is certainly not the chief source, as supposed by *Liebig*. This distinguished chemist, supposing (but here he is surely mistaken) that Maize, or Indian corn, does not contain any oily matter, has concluded that it must be from the fecula of this grain that cattle, fed upon it, derive the elements of their fat. The French chemists, on the other hand, maintain that Maize does contain oily matter in considerable quantity, and they are of opinon that this passes directly, and without having undergone any essential change in its composition, into the blood of the animals to which it is given for food.

This, and indeed most other questions of Vegetable Organic Chemistry, are still "sub judice;" and doubtless, it will require many and most patient inquiries to be made, before all the difficulties are satisfactorily solved.—*Annales de Chemie.*

---

### The Hæmospasic Method of Treatment.

Many medical men may perhaps not even know the meaning of the word *Hæmospasia*, although the merits of the practice, which it denominates, have been canvassed at great length some years ago in the Academy of Medicine, and valuable reports from the pens of MM. *Magendie* and *Serres* have been made, on two different occasions, upon the subject. The Monthyon prize has been twice awarded to the talented author of the discovery, M. *Junod;* and the Administrative Council of the Metropolitan Hospitals have expressed their marked acknowledgement of the great benefits which he has bestowed by his discovery on therapeutic medicine. He has uniformly proceeded with a truly scientific caution in introducing his new remedy to public notice. Not content with trying it on numerous occasions in his own practice, he has sought the opinion of his brother-practitioners, and solicited their frank and unbiassed judgment. In this respect, he has acted very differently from certain medical men of our times, whose only ambition seems to be to win popular applause, and a rich harvest of fees. The very quiet and unobtrusive course, which M. *Junod* has followed upon all occasions, will partly account for the comparatively little attention that has been paid to his ingenious discovery.

*Hæmospasia* (from αιμα sanguis, and σπαω traho, sugo), is a means of producing a powerful *revulsion* of the blood from one part, and an equally powerful *derivation* of the blood to another part, of the body, by removing the atmospheric pressure from a large extent of surface, as from one or both extremities at the same time. It is, therefore, so to speak, quite the same as dry-cupping; only on a large scale. "To produce an intense *raptus* of the blood from the deep-seated to the superficial parts of the body, to dissipate congestions, to counteract morbid fluxionary accumulations, and to relieve any organ or organs that may be oppressed with a surcharged circulation—such is the aim and object of the new therapeutic agent. By its means, we are enabled to withdraw, or displace, or accumulate, or concentrate a part of the mass of blood, according as the varying circumstances of the constitution, age, existing disease, and so forth, may render expedient."

In 1835, M. *Magendie* alluded to its great utility as a certain method of instantaneously attracting or *deriving* towards the extremities the blood, which would otherwise become congested in the viscera of the great cavities of the body;—and this, too, without causing any direct loss of the vital fluid.

That the remedy is one of no ordinary activity, is sufficiently evidenced by the immediate effects which it often produces. The face is rendered pale; the pulse becomes slower than it was before; at times there is a tendency to syncope; and often there is a good deal of disturbance of the gastric and intestinal function. All these symptoms are referrible to the powerful derivation of the blood from the heart and great bloodvessels towards the extremities of the body.

As patients usually express their feelings, under the operation of remedies, much better than any medical writer can describe them, it is always well to note such observations down at the time One said that, when the *hæmospasic* apparatus was applied, he felt as if he was quite deprived of his blood; another compared the sensation as if he had to bear the weight of ten foot-baths all at once; while a third observed that his life seemed to descend to his feet.

*Hæmospasic* revulsion has been employed with useful effects in a variety of inflammatory and congestive diseases. When the object of the physician is to divert the flow of blood to any internal part, and to relieve the accumulation, without having recourse to sanguineous depletion, no remedy is more worthy of trial than M. *Junod's* apparatus. How important is it to be able to do this in many cases, where the patient is of a delicate constitution, and where the system, if once much reduced, will inevitably be long of recovering its lost energies! Under such circumstances as these, the Hæmospasic method is a truly precious resource. The blood is withdrawn from the suffering part, and yet not a drop of it is lost. It has, however, been objected that, when the apparatus was removed, there would be forthwith a violent reaction, and that then the blood would be propelled with greater force than ever to the seat of the disease, so as perhaps to produce a more intense congestion there than had existed even at the first.

But this objection, though seemingly plausible, is contradicted by the results of experience: for, as the Hæmospasic injection or plethora takes place chiefly in the capillary vessels, the tumefaction, thereby induced, is found to subside very slowly and gradually. We have seen the apparatus applied in a great many cases, and have never yet observed the slightest inconvenience from its adoption in a single instance. As a matter of course, judgement and experience are as necessary for rightly using this, as they are for the employment of any other potent remedy. M. *Junod* having made no secret of his method, it has been indiscriminately recommended by not a few practitioners in this metropolis, who are ever on the look out for any novelty, and who are much more likely to be adepts at *drawing* the fees than the blood of their patients. We cannot do better than close these remarks with a paragraph from his little work, entitled *Methode Hæmospasique at Appareils du Docteur Junod.* 1843. *Paris*:—" It is not a question—and this remark I make most emphatically—of a secret remedy, of a mercenary industrialism, or of any mere speculation whatsoever. It is a remedial method, based on the acknowledged laws of animal physiology, and tested by numerous experiments and observations. I address myself only to men of education and practical knowledge, and solicit of them a patient and cordial examination of my assertions. With confidence I may say, alike to my professional brethren and to the patients, to the most censorious physician as to an enlightened public, come and see, examine and judge, for yourselves."—*Gazette Medicale.*

*Remarks.*—The Hæmospasic Method is, in our opinion, rather an ingenious suggestion than a useful remedy. In certain cases of chronic maladies, it may doubtless be used with advantage, just as any of the other medical novelties of the day not unfrequently are; but it is not likely ever to be so popular as they, from the inconvenience of its application, and the uncomfortable feelings which it often produces at the time. Let it not be supposed that we class M. *Junod* with the *puffing* doctors of the day—whether their panacea be cold water or steam baths, pills by the dozen or fractionary parts of a grain. He is far too honourable and honest a person to descend to anything unworthy of a professional gentleman. Nevertheless, we must protest against any laudation of his blood-attracting method in one or two diseases, in the treatment of which we find that it is recommended both by himself and others. For example, we are repeatedly told that it may supersede the employment of blood-letting in many cases of inflammation. What!—is there no other morbid element in this generic disease, save and except only the existence of a greater quantity of blood in the affected part than usual?—and is the human body so entirely a mere Hydrostatic machine that we can lessen or increase at will the actual quantity of blood in any part by simply regulating the amount of the atmospheric pressure upon it? Such a doctrine savours, we think, more of the 17th than of the 19th century; more of the times of *Borelli* than of those of M. *Andral.* Has not the latter gentleman, within the very last twelve months, been teaching his contrymen—for, thanks to British sagacity, the instruction was not required on this side of the Channel—that there is an intimate and essential change in the proportion of the constituents of the blood in all inflammatory diseases? The relative proportion of the fibrine is considerably higher than in health, while that of the red globules is but little, if at all, affected. If such be the case, does it not follow, as an obvious inference, that the merely diminishing of the actual quantity of blood in any part, can never reasonably be expected to cure an inflammation of that part? We have often—long before the publication of *Andral's* recent researches on Hæmatology—urged this argument, as one of decisive and insuperable force, against the Broussaian doctrine of recommending excessive bleeding in Typhoid fever, and other falsely-called inflammatory diseases. It is not the quantity of the blood, so much as its altered quality, that is at fault, alike in the Pyrexiæ and in the Phlegmasiæ; and it is only by paying due attention to the changes in the condition of the fluids, as well as of the vital forces which move them, that a safe and rational system of Therapeutics can be established. Such is the basis on which modern Humoral Pathology must be built.—*Rev.*

---

### MISCELLANEOUS NOTICES.

### *Utility of Calomel in Typhoid Fever.*

Drs. *Lombard* and *Fauconnet* of Geneva sum up an elaborate account of their experimental inquiries on this important point of practice in the following words:—

"Calomel diminishes the mortality of Typhoid fever, and renders all its symptoms less severe, more especially those that indicate a disturbance of the Nervous centres. It tends to abate the danger of any thoracic complications supervening, and, without being able to check them altogether, it causes them to be less serious in their consequences, as well as less frequent in their occurrence. It modifies and corrects in a very remarkable manner the condition of the alvine evacuations, and usually serves to diminish any diarrhœa if present, and to bring all the secretions to a more normal state. It rapidly cleanses the tongue, and renders it and the mouth less parched; it dissipates tympanitic distention and colicky pains of the bowels, and appears to exercise rather a serviceable, than an injurious, effect on the gastro-intestinal inflammation, which not unfrequently complicates Typhoid fever." Drs. L. and F. attribute the efficacy of calomel in this disease to its constitutional operation, rather than to any direct effects which it may have on the abdominal viscera. They remark that, there is almost always a decided amelioration of the symptoms, when the gums become slightly effected with the mercurial irritation.

(The observations of these gentlemen are in accordance with the general experience of most medical men in this country. Mercury seems unquestionably to exercise a very marked, and, in most cases, a very serviceable influence on the course of typhoid fever. Its *modus operandi* is probably twofold; *first*, by correcting and evacuating the secretions of the bowels; and, *secondly*, by altering and modifying the state of the circulating fluids, as well as the vital powers of the bloodvessels themselves. A favourite preparation with many practitioners is the Hydrargyrum cum cretâ, either alone or in combination with Carbonate of soda, Ipecacuan, Dover's powder, &c. The use of this mercurial, at stated intervals—in doses of four to eight grains, every six, eight, or twelve hours—with the exhibition of saline draughts between each dose, is, on the whole, by far the safest medication that can be resorted to in the majority of cases of low fever.)

### *Microscopic Examination of the Tartar of the Teeth.*

Hitherto this calcareous incrustation has always been regarded as a mere inorganic deposit from the saliva; but *M. Mandl* has been led, by his recent observations, to adopt a very different opinion respecting it. According to this gentleman, the tartar of the teeth is almost entirely composed of the skeletons or cases of infusory Animalculæ, agglutinated together by dried mucus; just as certain soils (so says the great German microscopic naturalist, *Ehrenberg*) consist of fossil Infusoria.

If a portion of the mucosity, which is adherent to the teeth, be mixed with a little water and heated, and then subjected to microscopic inspection, it will be found to exhibit a number of infusory Animalculæ, which are in active motion. Their shape is identical with that of the Animalculæ, known by the name of *Vibrio*. The presence of living Infusoria in mucosities was known to *Leuwenhock;* but the fact seems to have been forgotten, until revived by M. *Mandl.* Having satisfied himself that they exist in the mucosities of the mouth, he set himself to ascertain whether they contributed to the formation of the tartar of the teeth; and he was not long of discovering that this substance chiefly, if not entirely, consists of dead *vibrios* cemented together by dried mucus. From this circumstance, may we not infer that these Animalculæ are provided with an inorganic calcareous covering to their bodies?

It may be useful here to state that, according to the analysis of M. *Vauquelin,* the tartar of the teeth consists, in the 100 parts, of 66 of phosphate of lime, 9 of the carbonate, 14 of animal matter, and 3 of oxyde of iron and phosphate of magnesia.

*Medical Geography.*

" M. *Humboldt* has remarked, in reference to the botanical and pathological geography of the New World, that the highest limit of the *Melastomatous* plants is that also of the Yellow Fever. From this circumstance it may be inferred that the disease does not extend upwards beyond a certain elevation above the level of the sea. The Plague too obeys a somewhat similar law respecting its diffusion; for it is observed at Cairo that, while it is most prevalent in the low quarters of the city, it seldom affects the residents of the Citadel. At Constantinople, also, it very generally spares the Mountain of d'Alem-Daghe; and even the highest parts of the Seven Hills, on which this celebrated city is built, are generally exempt from it.

A similar remark is applicable to the *Endemic* Cholera. Not so with the *Epidemic* variety of this disease; this mounts much higher; and, as a certain degree of elevation above the level of the sea gives a diminution of temperature that is equal to a certain approximation to the Pole, it was not difficult to predict that this pestilence would not spare the colder regions of the earth. No locality seems to be quite exempt from its invasion. The basins of the Caspian Sea and Oral lake, the level of which is considerably lower than that of the ocean, suffered severely from its ravages; while, at the same time, it is well known that many of the loftiest residences in India have not escaped at different seasons.

With respect to the influences, which the nature of the soil of a country may exert on the development and diffusion of certain diseases, it may be stated that the Plague, like all marsh diseases, is generally most prevalent in argillaceous districts; that Phthisis and follicular Enteritis prevail in chalky, while Epidemic Cholera seems to be quite as common upon one geological formation as upon another. Goitre and Cretinism are found in chalky countries almost exclusively. In examining the Endemic diseases of any place, it is very necessary to attend to the character and qualities of the water that is drunk by the inhabitants. The water at Oran in Algeria is found to contain twenty times as much solid matter, dissolved or suspended in it, as the water of the Seine."

*Epizootic Gangrenous Angina.*

This disease occasionally proves very fatal among all sorts of cattle. It is much more prevalent, and also more malignant, during certain seasons, and in certain localities, than in others. When the food is of a bad quality, and especially when the water given to the cattle for drink is impure, the disease, when it breaks out, is always very severe and destructive. The symptoms vary a good deal in different seasons. There is often great prostration from the very commencement of the attack; and then the affection of the throat, and sometimes of the air-passages at the same time, is of a decidedly gangrenous character. In other cases, the symptoms are altogether of a more acute type: the pulse is full; the mucous surface of the nostrils and mouth exhibits a deep-red colour; the head, and more especially the ears, are very hot to the touch; and the animal evidently experiences much distress in swallowing. When the adynamic symptoms come on, the surface of the body and extremities becomes cold; the pulse is small and soft; and the expired air has an offensive smell. Sometimes a sanious discharge flows from the mouth and nostrils; the deglutition and breathing become more and more difficult; and death soon follows. The Necroscopic appearances usually found are these—infiltration of the subcutaneous cellular tissue of the head and neck; a softened decomposed condition of the mucous membrane of the mouth and throat, and sometimes even of the larynx and bronchi; and a dark pitchy and fluid state of the blood.

*Verminous Tumors in the Stomach and Bowels of the Horse.*

M. *Valenciennes,* in a Memoir recently read before the Academy of Sciences, describes two kinds of these tumors,—according as they are situated in the pyloric extremity of the Stomach, or in the Colon of the Animal. The entozoa in the one case are not the same as those in the other. The tumors are usually situated between the mucous and the fibrous membranes of the gut, and are provided with one or more openings, through which the entozoary animals may pass into its cavity. These perforations of the mucous lining are seldom associated with any other lesion of its texture. The tumors are generally separated, by numerous folds or partitions, into several compartments; these, however, communicate with each other, and there is always a greater or less amount of viscid mucus contained within. This mucus, in some cases, concretes and becomes so indurated that the tumor at length acquires quite a scirrhous consistence.

Verminous tumors seem to be of very frequent occurrence in the equine race; for of 25 horses examined by MM. *Rayer* and *Valenciennes,* they were found to exist in no fewer than 11.

*Tic Douloureux of the Face and Head.*

M. *Ducros* recently communicated to the Academy the details of some well-marked cases of this distressing disease, which were rapidly cured by the use of strong ammonia, applied to the palate, gums, &c. with a camel-hair brush, so as to occasion a profuse discharge of tears and saliva. He requested the physicians of several of the metropolitan hospitals to repeat his experiments on a large scale; and the results of their trials have been, he says, most satisfactory.

(The strong Aqua or Liquor Ammoniæ, taken internally, will be found to be a most valuable remedy in many cases of neuralgic suffering about the face and head, odontalgia, severe nervous headache, &c. The best mode of administering it is to mix from 20 to 40 drops in a cupful of very thick gruel, and to take this at bed-time, or whenever the paroxysm of pain is present. The ammonia must be well blended with the gruel, else it will irritate very painfully the inside of the mouth and throat. It should produce a profuse salivation and lachrymation. In very severe or obstinate cases, it may be applied outwardly at the same time.)

*The Epithelium-cells of the Intestines.*

MM. *Gruby* & *Delafond*—two of the ablest microscopists of the present day—announce, as the result of repeated observations, that " the Cellules of the Epithelium, which cover the villi of the intestines, are—when the animal is fasting—transparent, of an elongated shape, and contain an oval nucleus, which is slightly granular, while the cells themselves exhibit a translucent ring (*bouvrelet*) around it. During the act of chylification, these cells become much larger and more opaque; and within the ring, numerous minute molecules and globules are generally to be perceived. These molecules are transparent and resemble a good deal the globules of fat." These gentlemen subsequently describe the Epithelium-cells as having an aperture on their surface; this aperture being sometimes open, and at other times closed. They also describe a set of *Vibratile* bodies on the surface of the villi.

The villosities of the small intestines were found by them to have, in the living animal, a three-fold movement, which consisted in their being first elongated, then contracted, and lastly in being moved from side to side. This movement may be compared to that, which is often observed in Entozoary animals.

Each Epithelium-cell is believed to have a quadruple function—1, to fill itself with the crude chyle; 2, to divide and attenuate this chyle and to convert it into a pure homogeneous fluid: 3, to expel this purified chyle into the chyliferous vessel; and lastly to absorb other substances dissolved in the digestive juices, and convey them to the vascular apparatus. The functions therefore of these cells present a remarkable analogy with those performed by the lowest and most simple forms of animalcular existence; and it is not therefore very wonderful that some physiologists have actually regarded them as independent beings. (Many of the observations of MM. *Gruby* and *Delafond* agree perfectly with those of Mr. *Goodsir*, to which allusion is made in the first article of the present Number on Formative and Structural Anatomy.)

### *Proximate Cause of Hæmorrhage.*

M. *Andral*, in his recently-published Essay on Hæmatology, states it, as the result of his experimental researches, that some hæmorrhages are always connected with, if not immediately dependent on, a state of blood in which there is an excess of red globules; while others are very usually associated with an *hæmatosis*, characterized by a deficiency of the fibrine in the circulating fluid. The former are observed to occur in persons of a plethoric constitution; but they are far from being either as frequent, or as serious, as the second sort of the disease, such as takes place in scurvy and similar diseases. Is not this division of hæmorrhages very much the same as that adopted by the old writers, viz: into *active* and *passive* ?—a division that is incompatible indeed with certain theories of modern times, but one with which neither clinical medicine nor therapeutic instruction can dispense.

(The remark made here is quite just. There are several kinds of hæmorrhage, just as there are several kinds of fever; and it cannot therefore be wonderful that the same treatment is not appropriate in all. One case may require bleeding, and nauseating purgatives; while another demands the use of powerful tonics and astringents. The *internal* exhibition of alum is, we think, too much overlooked in the present day, as a powerful means of arresting many hæmorrhages.)

### *Maschaliatria, or Medication by the Axilla.*

Such is the new title of an old method of using remedies: it consists in merely depositing them in the axilla, and leaving them there to be gradually absorbed. Various medicines may be applied in this manner, either in the form of ointment, lotion, powder, &c. Accordingly, syphilitic affections have been thus treated with the blue ointment; agues with a pommade of quinine; and scabies with sulphur ointment. Professor *Forget* of Strasburg tells us that he has treated many cases of these diseases successfully in this manner. He proposes to try various other remedies according to the Maschaliatric (μασχαλη axilla, and ιατρος medicus) method; such as opium and nitrate of morphia, digitalis, strichnine, ioduret of potassium, either mixed with some unctuous substance, or in a state of solution. The cutaneous absorption in the axilla, he tells us, is more active than in almost any other part of the body. (A truly French proposal!)

### *Memoranda on Cutaneous Diseases.*

M. *Emery*, one of the physicians of St. Louis Hospital, has of late been very strongly recommending the use of Pitch, internally as well as externally, in cases of Lepra, and of Styrax balsam in certain cases of Lupus. Neither suggestion, however, can claim the merit of any originality. During the clinical attendance of M. *Alibert*, pitch was successfully used in a great variety of chronic skin diseases, such as Porrigo, Favus, Lepra, Impetigo, and even in Scabies; and, upwards of ten years ago, Dr. *Dauvergne* published a paper in the Bulletin de Therapeutique, pointing out the good effects of Styrax balsam in certain esthiomenous or corroding ulcers. This gentleman has lately recommended a solution of the sulphate of iron as an admirable application in cases of Sycosis, Mentagra, and Gutta rosea. Sometimes he blends the powdered sulphate with charcoal, and applies it to the excoriated surface in the dry state. The internal remedies which he generally uses at the same time, are bleeding and purgatives in plethoric subjects, and the ioduret of potassium, when all symptoms of inflammatory irritation have been removed. (The Liquor Potassæ—given in doses of from 15 to 30 drops, three times a day—is an admirable remedy in many cases of inveterate skin disease. According to our observations, it is far more efficacious, and perhaps, too, less injurious, than the potash in combination with iodine. The Liquor potassæ may be given in milk, beer, decoction of Sarsaparilla, &c.

With respect to the sulphate of iron as an external application, we cannot believe that it possesses any curative virtues above those of the Sulphate of Zinc, or of the Sulphate of Copper, that are in daily use. The White Vitriol is our favourite; and the best way of applying it is by dipping rags of soft linen in a tepid solution of the salt, and covering these with a piece of oil-skin. If used thus, the Lotion will not require renewal oftener than night and morning. In some cases, a little Hydrocyanic acid may be conveniently added to the Solution with advantage.)

*Treatment of Rheumatism.*

Two very different remedies are highly praised, at the present moment, by some of the French physicians, in the treatment of this very common disease. These are, the Sulphate of Quinine and the Nitrate of Potash. M. *Briquet*, and a host of followers, descant, in terms of no measured commendation, on the virtues of Quinine; while M. *Martin Solon* is no less energetic in his laudations of his favourite. Neither of these gentlemen seems to be at all imbued with Homœopathic principles; as far at least as regards the doses which they administer; for the one is in the habit of giving from 15 to 20 grains of Quinine, and the other from half an ounce to an ounce and a half of the Nitre, at a time. We need scarcely say that we utterly disapprove of the indiscriminate practice of both.

Our chief motive for alluding to this subject at present, is to introduce the following sensible remarks by a writer in the Gazette Medicale, in reference to Dr. *Solon*'s Memoir. " No one has ever questioned the perfect accuracy of the assertions made by this gentleman. As far as we can judge, we should say that the Nitrate of Potash, administered in large doses, is not only inoffensive, but is often very useful. There is, however, in all the statements and reports that have been made on the subject, an important blank which we should have wished to have seen filled up. Be it remembered, that not a few cases of Rheumatism have a natural tendency to relieve themselves by perspiration, if the fever do not run high, and the pains are not very severe; whereas, the disease is apt to be obstinate and unyielding, when the symptoms are otherwise. It certainly does seem to us that many of the cases, related by Dr. *Solon*, might have got well if nothing had been given, except simple warm drinks.

" This remark applies with equal force to every other remedy that has, at different times, been recommended for the cure of Rheumatism, as well as to the Nitrate of Potash. It requires, therefore, much more discrimination than is usually imagined, to determine with accuracy the genuine therapeutic effects of medicines."

Perfectly true. The credit, that properly belongs to the *vis medicatrix naturæ*, is not unfrequently awarded to the medicines that are administered.

### *Medical Anecdote.*

An old physician, on being asked his opinion of a new remedy, that was highly praised for its extraordinary virtues in a certain disease, very gravely replied,—"Depechez-vous de vous en servir, pendant qu'il guerit." A severe, but well-merited censure on the fond credulity, not of the public only, but also of many medical men. Hydropathy is curing vast numbers at present: how long will it continue to do so? Brandy and salt were in favour a twelvemonth ago; what has now become of them?—Veratria was a sovereign remedy against neuralgia a few years back, and the chemists were making a thriving trade of its sale at sixpence a grain: now, it is seldom asked for. Unfortunately, there always seems to be an eager desire for the discovery of something new; as if there were not remedies enough, if we but knew *when* and *how* to use them. The clever workman is seldom changing his tools. It is true, in medicine as in dress, that often "there is nothing so new as that which is forgot." Many of the vaunted discoveries and inventions of the present day have been found to be but the resuscitations of what was long neglected or perhaps concealed.

### *Statue to Bichat.*

A statue has recently been erected in honour of this distinguished physiologist in his native town of Bourg (Ain), and the ceremony of inauguration was performed, on the 25th of August last, with "un eclat vraiement extraordinaire." The Academy of Medicine was represented by its Secretary, M. *Pariset;* the Faculty of Medicine of Paris, by M. *Royer-Collard*; that of Strasbourg, by M. *Forget;* Military Surgery, by M. *Hippolyte Larrey;* the medical men of Marseilles, by M. *Roux;* those of Lyons, (the cradle of *Bichat's* studies), by MM. *Brachet*, *Barrier*, and eight or ten other gentlemen. As a matter of course, many eulogistic discourses and orations were pronounced on the occasion.

The statue represents *Bichat* studying the phenomena of life on the body of a young child; there is a half-dissected corpse stretched out at his feet. (A strange and most unseemly design surely! Has the illustrious Anatomist a scalpel in his hand?)

### *On the Cutaneous Affection called Molluscum.*

The name given to this rather rare skin disease, is derived not from any resemblance which it bears to certain warts, tumors, or excrescences, but solely from it being supposed to have been brought from the Molusques (Mollucca) Islands, where one form of it—which was described by Bontius in the 17th century—prevails epidemically. Most writers have described three sorts or species of it; viz.—1, the Endemic and contagious Molluscum of Amboyna; 2, the Sporadic and non-Contagious m.; and, 3, the Atheromatous m. described by *Bateman.* M. *Gibert* adds a fourth, which he calls Stearic m. A case of this variety he narrates at considerable length. It occurred in a patient, who was affected with chronic Jaundice at the same time. On different parts of the skin, there were numerous hard projections or knobs, sessile, indolent in character, and of a whiter colour than the rest of the skin. On examining them first with a strong magnifying-glass, and afterwards with various chemical re-agents, M. *Gruby* concluded that they were composed of a greasy substance, analogous to *Stearine,* deposited under the epidermis, and in the meshes of the true skin. Their size varied from that of a pin's-head, to that of a good large pea, and upwards. One, that was almost as large as a hazel-nut, was removed with the knife: it was found to consist of a firm white substance, composed of globules that were soluble in æther, and not affected by acids.

M. Gibert saw another case, very similar to this one, so far back as the year 1829: the first patient too was affected with jaundice. From the circumstance of the co-existence of hepatic disease in both instances, M. *Gruby* suspects that the two affections are related to each other, in the way of cause and effect; and that the *cholesterine,* that ought to be eliminated in the bile, becomes converted into the fatty matter that is found deposited in the cutaneous tissue. M. *Gibert* closes his observations by remarking that "the genus Molluscum comprehends several species, which have no characters in common with each other except these —cutaneous excrescences that are indolent, of a firm consistence, having nearly the same colour as that of the skin, and geneιally incurable."

*The Bisulphuret of Carbon.*

This compound has been used (with decided benefit, it is said) by many of the German physicians internally in cases of Rheumatism, Neuralgia, chronic Gout, &c. and externally in burns and some cutaneous affections.

M. *Otto* of Copenhagen, recommends it very highly; the usual dose which he gives, is four drops of a mixture, composed of one part of the Bisulphuret and two parts of alcohol, given every two or three hours.

(We are not surprised that any compound of Sulphur should be serviceable in many cases of chronic rheumatism and neuralgia, as the simple element itself has no ordinary efficacy in all such complaints. A tea-spoonful of brimstone in a small cupful of milk, taken every night at bed-time for a week or two together, is one of the best of all remedies, that we know of, against old obstinate rheumatic aches, cramp of the legs, the pains that are connected with a varicose state of the veins, chronic sciatica, &c.

The well-known nostrum—the "Chelsea Pensioner"—that has so long had high repute in chronic rheumatism, is mainly indebted to sulphur for its virtues. It may be worth while to mention its composition. It is made thus :—

℞ Flor. Sulphuris ℥ij.

Pot. Supertartrat. ℥j.

P. Guiaci ʒj.

P. Rhei ʒij.

Spir. Nucis Mysistic. ʒij.

Mellis q. s. ut fiat electuarium.

The dose, one or two drachms every morning and evening.)

*A Royal Letter of Thanks.*

The following very gracious epistle was recently sent by the King of Prussia to M. *Achille Compte,* a very able naturalist of France.

"I have received, Sir, with very particular satisfaction, the great work on Natural History which you have been pleased to transmit to me by Baron Humboldt. It cannot fail, not only to benefit science and public instruction, but also to render accessible, by means of its admirable drawings, the researches which, in the steps of the illustrious Cuvier, have shed a new light over the entire field of Animal Organisation.

"In thanking you for your present, I am pleased to transmit to you, as a token of my satisfaction, the large medal awarded for distinction in the sciences.

"I pray, M. Achille Compte, that God may keep you in His most worthy and holy care.

"FREDERICK WILLIAM.

"*Sans Souci,* 23*rd August,* 1843."

